# Atomic-Level and Surface Structure of Calcium Silicate Hydrate Nanofoils

**DOI:** 10.1021/acs.jpcc.3c03350

**Published:** 2023-09-08

**Authors:** Ziga Casar, Aslam Kunhi Mohamed, Paul Bowen, Karen Scrivener

**Affiliations:** † Laboratory of Construction Materials, Institut des Matériaux, Ecole Polytechnique Fédérale de Lausanne (EPFL), CH-1015 Lausanne, Switzerland; ‡ Institute for Building Materials, Department of Civil, Environmental and Geomatic Engineering, ETH Zürich, CH-8093 Zürich, Switzerland

## Abstract

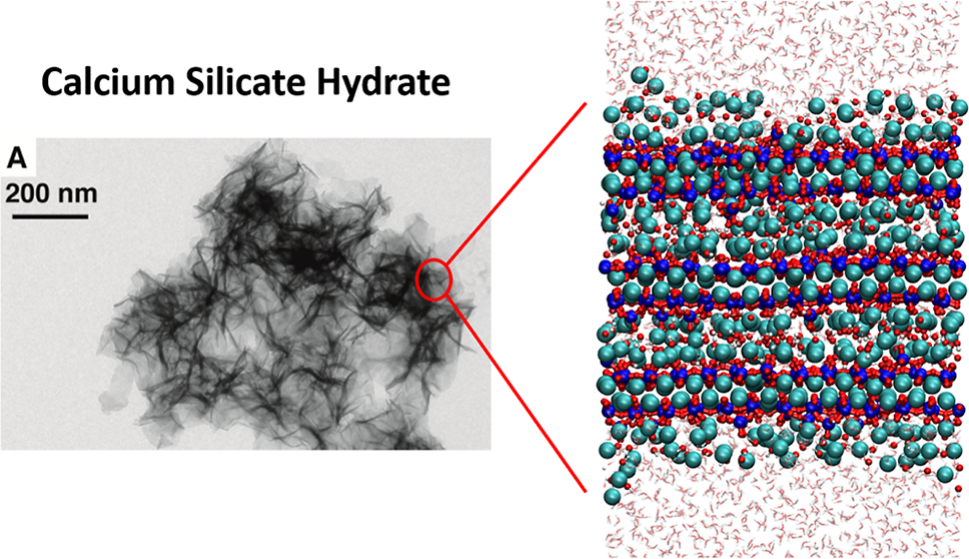

Deciphering the calcium silicate hydrate (C-S-H) surface is crucial for unraveling the mechanisms of cement hydration and property development. Experimental observations of C-S-H in cement systems suggest a surface termination which is fundamentally different from the silicate-terminated surface assumed in many atomistic level studies. Here, a new multiparameter approach to describing the (001) basal C-S-H surface is developed, which considers how the surface termination affects the overall properties (Ca/Si ratio, mean chain length, relative concentration of silanol and hydroxide groups). Contrary to current beliefs, it is concluded that the (001) C-S-H surface is dominantly calcium terminated. Finally, an adsorption mechanism for calcium and hydroxide ions is proposed, which is in agreement with the surface charge densities observed in previous studies.

## 1 Introduction

The production of cementitious materials is responsible for 5–8% of anthropogenic CO_2_ emissions.^1^ With the rising world population and the related needs for infrastructure, the demand for cement will increase. To lower the carbon footprint of cement, a fundamental understanding of the chemistry at the phase interfaces is needed.^2^

The main hydration product of Portland and blended cements is calcium silicate hydrate (C-S-H). C-S-H forms around 50 to 60% by volume of hardened cement paste and is the primary binder that binds together other crystalline hydration products and aggregates and gives cohesion to the material.^3^ Nucleation and growth of C-S-H are the underlying mechanisms behind the main hydration stage, occurring between a few hours and 1 day.^4^ C-S-H forms a continuous nanoporous network that reduces the transport of ions, such as chloride,^5^ which causes durability issues in steel-reinforced concrete structures.

**Figure 1:**
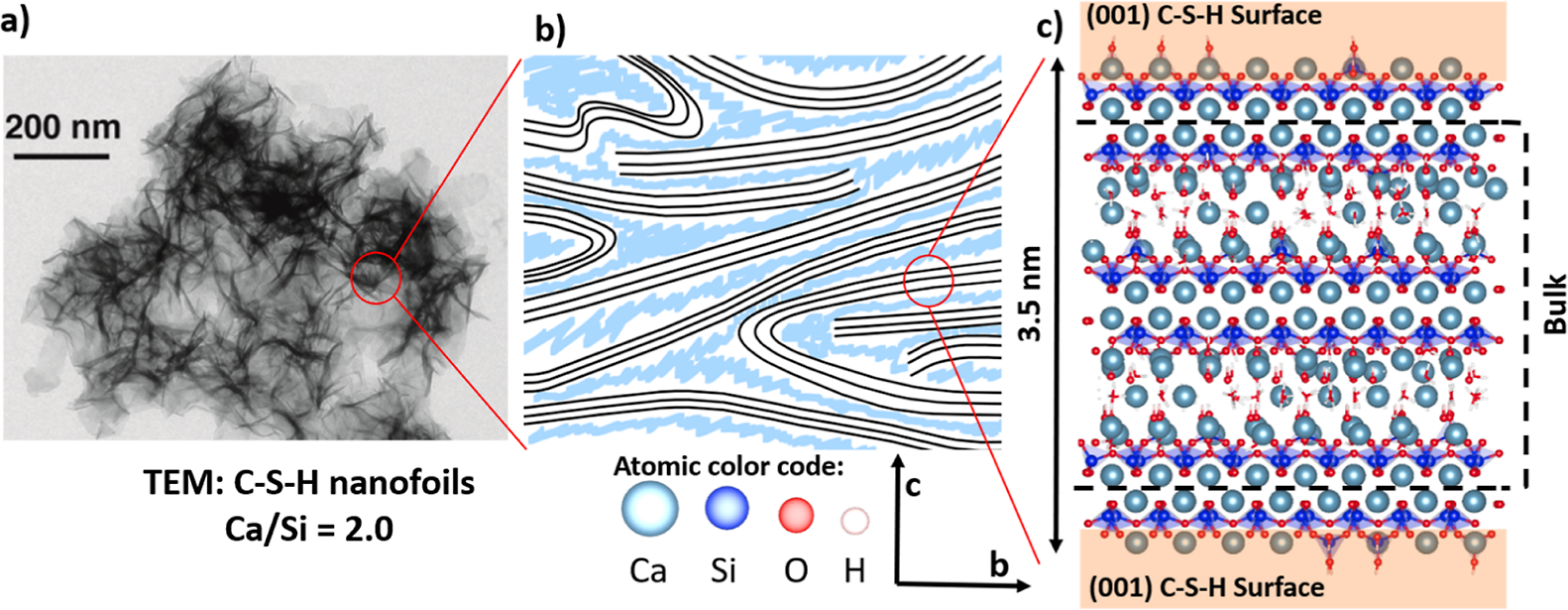
C-S-H nanofoils: (a) TEM image of synthetic C-S-H with Ca/Si = 2.0 (reproduced from Kumar et al.^8^ Copyright 2017 American Chemical Society) (b) schematic representation of the C-S-H nanoporous network (black lines represent the individual calcium-silicate sheets and blue represent water), and (c) computational model of 3 layers, 2 interlayers thick Ca/Si = 1.7 C-S-H nanofoil with the dominant (001) basal surfaces.

Transmission electron microscopy (TEM) images of C-S-H from samples of hydrated tricalcium silicate (C_3_S)^6,7^ and TEM images of high Ca/Si pure phase synthetic C-S-H^8,9^ show a nanofoil morphology, as seen in Figure 1. The nanofoils have a length around 100–200 nm^8,10−12^ and a thickness below 5 nm.^10,11,13,14^ The measured specific surface area is in the range between 200 and 300 m^2^/g.^15−19^ These observations underline the importance of C-S-H surfaces.^20^

The bulk structure of C-S-H is reasonably well understood.^3,8,21,22^ C-S-H can be considered as a highly defective 14 Å tobermorite with variable chemical composition and structure. While there is no long-range order, X-ray diffraction patterns give broad C-S-H peaks, which can be attributed to atomic distances within the calcium-silicate sheets.^9,14,23^ The calcium-silicate sheets are made of calcium oxide layers to which silicate chains are attached on either side. These sheets are separated by interlayers containing water molecules and ions (Figure 1). Contrary to 14 Å tobermorite, which has a Ca/Si ratio of 0.83, C-S-H has a variable Ca/Si ratio of 1.2 to 2.1, with an average of 1.7 in plain Portland cements at early ages. In 14 Å tobermorite, the silicate chains are composed of a dreierketten structure with silicate dimers, which are linked with bridging silicate tetrahedra Q^2b^ (see Si-Surface 2 in Figure 3).^24^^29^Si nuclear magnetic resonance (NMR) experiments on phase pure high Ca/Si C-S-H show the prevalence of the Q^1^ end-of-chain dimeric silicate species,^8,25,26^ which is a consequence of missing Q^2b^ silicates. Kumar et al.^8^ showed that most of the bridging sites are occupied with calcium ions instead, which contributes to high Ca/Si ratios in C-S-H. At Ca/Si = 1.75, only 30% of bridging sites are occupied with a Q^2b^ silicate and the remaining 70% with calcium ions.^26^ The calcium in these bridging sites is stabilized by an environment of strong hydrogen bonding.

The ratio of Q species is used to derive the mean chain length (MCL).^27^ Assuming a dreierketten structure and absence of silicate monomers,^8,27^ an MCL = ∞ represents the 14 Å tobermorite with no missing Q^2b^ silicates, an MCL = 2 would be a structure with no Q^2b^ silicates, and at Ca/Si = 1.75, the calculated MCL ranges from 2.55 to 2.89.^8,26^^29^Si NMR shows the complete absence of any Q^3^ silicate,^8,26^ making it clear that there is no connectivity between silicates of opposite silicate chains via the interlayer, as observed in 11 Å tobermorite.^28^ Other studies have found an increase of hydroxide groups and a decrease of silanol groups with increasing Ca/Si ratios.^3,22,29^ All these findings indicate that the high Ca/Si ratios observed can be attributed to the calcium substitution of the bridging Q^2b^ silicate and the addition of Ca^2+^ and OH^–^ ions in the interlayer.

In contrast to the bulk, the atomic structure of the surface of C-S-H is poorly characterized. Previous studies of interactions with C-S-H surfaces are summarized by Duque-Redondo et al.^3^ and Kunhi Mohamed et al.^30^ In most previous studies, authors have considered the energetically most favorable tobermorite basal (001) surface^31^ to have a Q^2b^ silicate termination (Si-Surface 2 in Figure 3). Due to the low Ca/Si of 14 Å tobermorite, some authors introduced defects to raise the Ca/Si ratio up to 1.4.^32−34^

The present paper shows how the brick model^21^ combined with results from experimental measurements (Ca/Si ratio, MCL, relative concentration of silanol and hydroxyl groups) can be used to construct pragmatic models of realistic C-S-H surfaces. By considering the C-S-H structure characteristics, a correlation between system properties and the surface silanol density is found. A mechanism for calcium and hydroxide adsorption is proposed, which gives excellent agreement with previous studies on surface charge densities. Finally, a full atomistic model of the C-S-H nanofoil structure is proposed.

## 2 Methods

Sections of C-S-H nanofoils were modeled as periodic structures in the *a* and *b* axis directions and with water molecules on both sides of the basal (001) surface in the *c* axis direction (see Section S1). The (001) surface is parallel to the calcium-silicate sheet.^31^ The C-S-H sections were generated with the brick model from Kunhi Mohamed et al.^21^ The model allows a systematic introduction of defects into the 14 Å tobermorite structure from Bonaccorsi et al.,^24^ to give C-S-H bulk structures with representative Ca/Si ratios, consistent with experimental data.^3^ The model is gaining acceptance^3,35−38^ and can be used to construct C-S-H surfaces, which can then be used for molecular simulations of adsorption processes.^39^ One main advantage of the brick model is the alphanumeric code (notation), which means the structure is exactly described.^3^ The details on model construction can be found in the Supporting Information.

The in-house code for generating structures according to the brick model positions the atoms in a predetermined way. This can result in initial atomic positions which can be far from the minimum of the potential energy surface. Therefore, a suitable equilibration protocol was used. First, a quasi-minimization step was carried out, followed by an isothermal-isobaric (*NPT*) run to relax the simulation box and achieve a bulk water density of approximately 1 g/cm^3^. Afterward, the system was heated to 700 K in the canonical (*NVT*) ensemble to ensure enough energy to reach a more stable minimum before the production run. The final production run was carried out in the *NPT* ensemble at 300 K and 1 bar with a timestep of 0.28 fs for at least 20 ns. All simulations were carried out with LAMMPS^40^ and a modified version of the CemFF2 force field.^25,41^ CemFF2 (and Erica FF2) use the adiabatic core–shell model^42^ for the polarizability of silicate oxygens, which is needed for the right prediction of structural properties of calcium silicate hydrates.^41,43^ The force field uses harmonic angles and formal charges of the silicates; therefore, it does not overpredict the electrostatic forces. An overprediction of the electrostatic forces would result in higher adsorption of cations on the negatively charged silicates,^44^ as can be the case for the popular ClayFF^45^ and CSH-FF^46^ force fields. For further reading on interatomic potentials and the advantages of polarizable force fields for silica and related systems, the reader is referred to the excellent review paper of Müser et al.^47^ The calcium–water interaction was adopted from Mamatkulov et al.,^48^ which has proved reliable in adsorption studies on charged amorphous silicate surfaces.^44^ A detailed description of the simulation protocols and force field parameters, as well as structure files, are provided in the Supporting Information (Sections S2 and S3).

The experimental results which were used in conjunction with the brick model are (i) X-ray fluorescence (XRF) and inductively coupled plasma spectrometry (ICP-OES) for the measurement of the Ca/Si ratio;^9^ (ii) ^29^Si NMR for the quantitative analysis of different silicate species (Q-sites),^8,25,26^ which are usually expressed as the MCL; and (iii) IR and Raman spectroscopy for the relative concentration of silanol (Si-OH/Si) and hydroxide groups (Ca-OH/Ca).^3^ All of these experimental methods measure overall properties, not just the bulk, but, including the surface. The origin of experimental data is elaborated on in Section S1.1.

Since C-S-H exists only at high pH, a 90% deprotonation of surface silanol groups was assumed for the calculation of the characteristics of the structures (Ca/Si ratio, MCL, relative concentration of silanol and hydroxide groups). This deprotonation level agrees with the value for C-S-H in calcium solution at pH 13, as predicted by grand canonical Monte Carlo simulations.^49^ The experimentally observed characteristics for the C-S-H nanofoil models were taken from Duque-Redondo et al. (Table 2).^3^

## 3 Results

**Figure 2:**
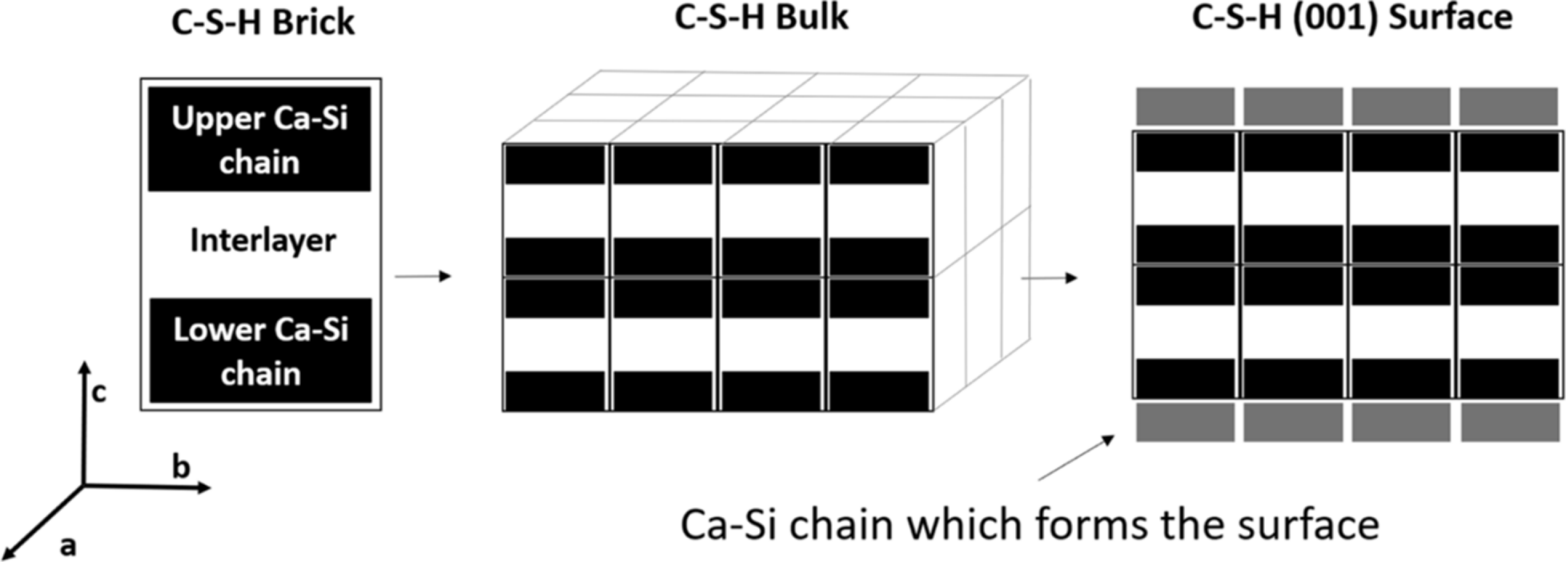
Schematic representation on how to construct the C-S-H (001) surface with the building brick description.^21^

Using the brick model from Kunhi Mohamed et al.,^21^ a basal C-S-H (001) surface can be constructed as follows (Figure 2). First, the size of the system needs to be decided; for example, a 4 × 4 × 2 system would mean that a structure which measures 4 C-S-H bricks (defective building blocks of tobermorite 14 Å unit cells) in the *a*-axis direction, 4 in the *b*-axis direction, and 2 in the *c*-axis direction is generated, creating a structure which is 2 interlayers thick. Second, defects need to be selected which will be introduced into the C-S-H bricks. These defects need to be chosen with care so that the overall bulk properties mimic the experimentally measured properties (Ca/Si ratio, MCL, relative concentration of silanol and hydroxyl groups). The defects which can be used are described in ref (21). Third, Ca–Si chains are added to the top and bottom of the bulk structure in order to generate the C-S-H nanofoil with the (001) surface. For a detailed description, we refer the reader to the Section S1 of the Supporting Information.

### 3.1 Thickness and Surface Area

In the *c*-direction, the C-S-H bulk structure is flanked by two (001) surfaces (Figure 2). The bulk structure has a Ca/Si of 1.7. The examined structures differ from each other by the number of calcium-silicate layers (Table 1).

**Table 1:** Calculated Specific Surface Area and Thickness as a Function of the Number of Interlayers^a^

number of layers (number of interlayers)	approximated specific (001) surface area [m^2^/g]	approximated thickness of the C-S-H nanofoil [nm]
1 (0)	870	0.8
2 (1)	360	2.2
3 (2)	220	3.5
4 (3)	160	4.9
5 (4)	130	6.3
exp	200–300^16,17,19^	3.5–4.0^10,14^

a The thickness is measured between surface Q^2b^ silicates, and it does not include adsorbed ions.

The C-S-H structure with 3 layers, 2 interlayers (Figure 1) measures 3.5 nm from the Q^2b^ bridging silicate on one surface to the Q^2b^ silicate on the other surface. For comparison, the structure with 4 layers, 3 interlayers measures 4.9 nm in thickness. The experimentally measured thickness for C-S-H with Ca/Si = 1.0 is 3.5 nm^14^ and calculated 4 nm^10^ for Ca/Si = 2.0. Adsorbed ions were not considered in the present model.

To verify the choice of thickness for the computational model, the surface area as a function of the number of layers was calculated. Since the exact atomic composition of a computational atomistic system is known, it is possible to calculate the mass of the system and therefore its specific surface area as SSA = area/mass. In Table 1, the approximate specific (001) surface area for the (001) surface model systems as a function of the number of layers is reported, whereby one (1) layer corresponds to a C-S-H nanofoil with a single layer and no interlayers. The calculated specific surface area is only for the (001) surfaces and neglects other surfaces, such as the lateral (100) surfaces, and is therefore slightly underestimated.

As seen from Table 1, the 3 layers, 2 interlayers C-S-H model results in SSA of approximately 220 m^2^/g, which is consistent with experimental observations. It can be concluded that the C-S-H nanofoils are indeed about 100–200 nm long,^8,10−12^ and only 3 layers, 2 interlayers thick, which results in a thickness of 3.5 nm.

### 3.2 Surface Termination

**Figure 3:**
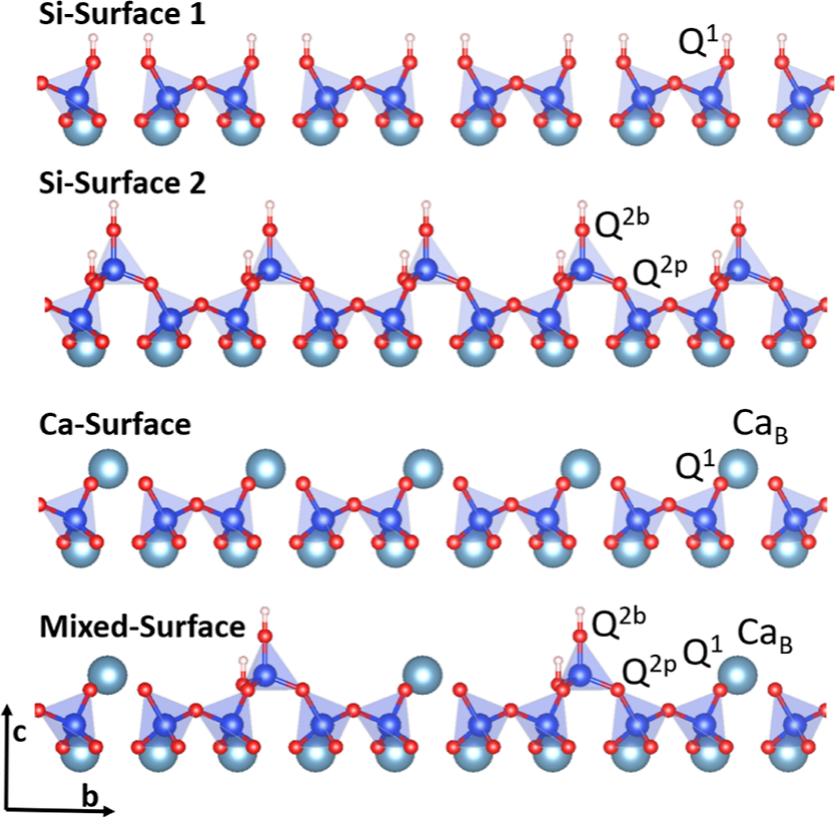
Investigated surface terminations. Color legend same as in Figure 1.

Due to the narrow thickness of C-S-H nanofoils (3 layers, 2 interlayers), the surface-associated calcium-silicate chains represent one-third (1/3) of all calcium-silicate chains. Therefore, it is expected that the surface termination will play a vital role in the overall characteristics of the structures (Ca/Si ratio, MCL, relative concentration of silanol and hydroxide groups). Four different (001) surface terminations were considered (Figure 3):

- Si-Surface 1: only Q^1^–Q^1^ dimers exist. All bridging sites are unoccupied. Each Q^1^ has one silanol group, resulting in a surface silanol density (SSD) of 4.8 OH/nm^2^
- Si-Surface 2: dimers are linked with bridging silicates Q^2b^. Silanol groups are only found on Q^2b^ silicates (two silanol groups per Q^2b^). SSD = 4.8 OH/nm^2^
- Ca-Surface: dimers are linked with bridging calcium ions—Ca_B_. No silanol groups on the surface. SSD = 0 OH/nm^2^
- Mixed-Surface: dimers are linked with either Q^2b^ or Ca_B_. Silanol groups can be found only on Q^2b^ silicates (SSD < 4.8 OH/nm^2^), as for the interlayers.

As can be observed in Figure 3, the considered surface terminations result in very different local Ca/Si ratios and MCL. The Si-Surface 2 has the lowest Ca/Si ratio (0.66) and longest MCL (∞), while the Ca-Surface has the highest Ca/Si (1.5) and lowest MCL (2). It is expected that a large difference in the observed structural characteristics of the surface and the bulk would greatly influence the overall characteristics of the C-S-H nanofoil.

**Figure 4:**
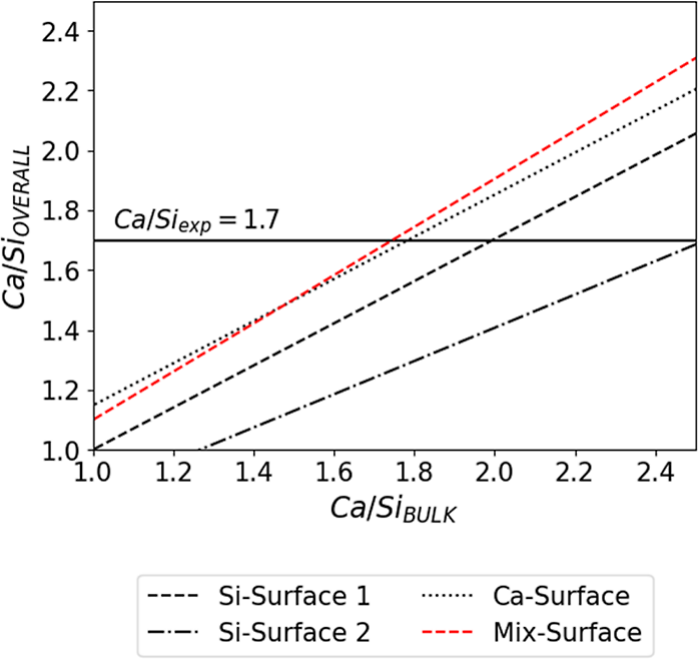
Correlation between the overall Ca/Si ratio on the Ca/Si of the bulk structure for the 3 layers, 2 interlayers C-S-H structures with given (001) surface terminations.

**Figure 5:**
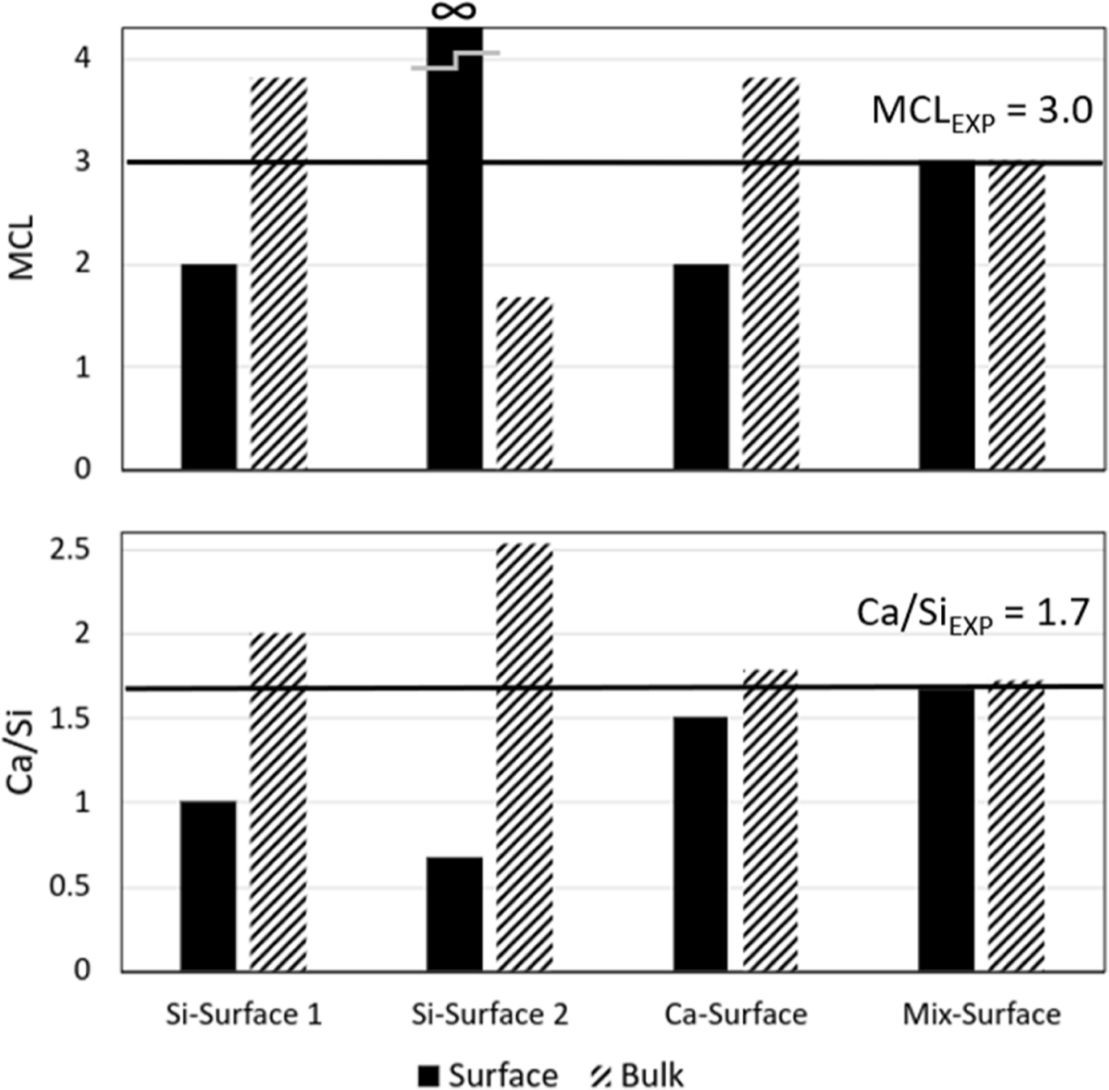
MCL and Ca/Si of the surface and bulk for C-S-H nanofoils with Ca/Si = 1.7 and selected surface terminations. The horizontal line represents the targeted experimental values.

First, considering the most widely assumed Si-Surface 2.^31,34,35,50^ As seen in Figures 4 and 5, to reach an overall (entire nanofoil) Ca/Si of 1.7, the Ca/Si of the bulk C-S-H structure would need to be 2.5. This is due to the low Ca/Si ratio of the surface (Ca/Si_surf_ = 0.66). The surface silicate dimers are always linked with Q^2b^ silicates (MCL_surf_ = ∞), therefore the theoretical MCL of the bulk would need to be 1.76. MCL of 2 represents silicate chains with all Q^2b^ silicates absent. For MCL lower than 2, the structure would need to have missing Q^1^ silicates in the Q^1^–Q^1^ dimer pairs. If one Q^1^ of the dimer is absent, the remaining silicate species is not connected to any other silicate, and it would appear as Q^0^ species in the ^29^Si NMR spectra. Experimental ^29^Si NMR of phase pure synthetic C-S-H does not show any Q^0^ peaks.^8,25,26^ Thus, it is reasonable to assume that such a surface termination of the C-S-H nanofoil with Ca/Si of 1.7 is not possible.

The Si-Surface 1 and Ca-Surface have the same structure of the silicate chains. Due to the absence of Q^2b^ silicates, the corresponding surface MCL is then 2.0. Therefore, a bulk structure with a high MCL is needed (MCL_bulk_ = 3.74). Due to the calcium in the bridging site (Ca_B_), the surface Ca/Si of the Ca-Surface equals 1.5, while for the Si-Surface 1 equals 1.0. The MCL decreases with an increase in the Ca/Si ratio.^3,27^ It is contradictory to have different Ca/Si in the bulk (2.0 for Si-Surface 1 and 1.79 for Ca-Surface) while maintaining the same MCL. The lower Ca/Si difference between the surface and bulk would make the Ca-Surface termination more likely than the Si-Surface 1 termination. However, Ca_B_ fulfills the charge neutrality of the bridging site as well as the surface in general, which is contrary to the experimental observations which suggest high surface charge densities.^16,17,49,51^ Finally, due to the lack of surface silanol groups, the Si-OH/Si ratio of the bulk would be required to be 13%. Assuming again that silanol groups are preferentially found on Q^2b^ silicates, this would result in 41% of non-sharing bulk Q^2b^ oxygens to be protonated. Kunhi Mohamed et al.^21^ showed that silanol groups become energetically less favorable as the Ca/Si ratio increases, which is also supported by the falling Si-OH/Si ratio.^3^

Assuming the C-S-H nanofoil grows in the *b*-axis direction, in the direction of silicate chains. Therefore, all silicate chains grow from the same solution. There is no obvious reason why the silicate chains on the surface should be different from the ones in the bulk in terms of MCL. In order to minimize the discrepancy between the surface and bulk, the Mixed-Surface was constructed with a MCL_surf_ of 3.0. Simultaneously, a bulk MCL with 3.0 is achieved. To obtain an overall Ca/Si of 1.7, a bulk Ca/Si of 1.91 is needed. However, due to the required bulk Si-OH/Si of 11% (overall Si-OH/Si of 9%), such a high Ca/Si ratio of the bulk is unlikely.^21^ Therefore, a bulk with Ca/Si of 1.72 was chosen, which results in an overall Ca/Si of 1.48. The Ca/Si ratio can be increased with calcium adsorption onto the surface. In dropwise precipitation of a pure phase with a high Ca/Si ratio C-S-H, a discrepancy between thermodynamically calculated and measured calcium concentrations in solution is observed.^9^ The difference increases with the increase in the Ca/Si ratio. This finding suggests that the amount of adsorption increases with increasing Ca/Si. To achieve the desired Ca/Si of 1.7, four Ca^2+^ ions need to be adsorbed per surface Q^2b^ site.

While there are a wide variety of possible surface terminations, it is evident that the MCL is the first characteristic to be considered when defining a C-S-H nanofoil model. The differences between the overall and bulk characteristics decrease with increasing thickness (the number of layers). However, the MCL remains dominant for the construction of the C-S-H nanofoil model (Section S1.3). Since C-S-H can be considered as a defective tobermorite structure, the surface termination of a mixed type (Ca_B_ and Q^2b^) is a logical choice. Finally, the experimental evidence suggests a high calcium affinity at the surface, with which the right Ca/Si can be achieved and is discussed in the next sections.^9^

### 3.3 Surface Silanol Density

Minerals immersed in an aqueous solution undergo a pH-dependent protonation of surface oxygens and deprotonation of chemisorbed water molecules, therefore developing a surface charge.^52^ In calcium silicate hydrate, silanol groups can only be found on non-sharing oxygens of Q^2b^ silicates. This is supported by the declining amount of silanol groups with the decrease in MCL.^3^ Since the MCL represents the ratio between Q^2b^ and Q^1^ silicates (Supporting Information 1), it can be used to express the surface silanol density (SSD)
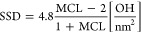
1

It is evident that lower MCL values (higher Ca/Si) correspond to lower surface charge density. From eq 1, one can calculate the SSD for the Si-Surface 2 (MCL = ∞) to be 4.8 OH/nm^2^, since . This is the reported value for the Si-Surface 2 termination in the literature.^49^ At the opposite limit of MCL = 2 (Ca-Surface), the calculated SSD equals 0 OH/nm^2^ since no Q^2b^ silicates are present on the surface. The studied surface termination (Mixed-Surface) with MCL = 3 corresponds to SSD of 1.2 OH/nm^2^.

### 3.4 Calcium Adsorption

High SSDs result in high (negative) surface charges, which should be balanced by cation adsorption. Calcium ions have a tight hydration shell, which does not allow for water molecules to shift within the hydration shell, therefore favoring outer sphere adsorption.^44,53,54^ The MD simulations carried out (see Supporting Information 5) on the 90% deprotonated Si-Surface 2 reveal that, on an average, around 14% of the Ca^2+^ becomes inner sphere adsorbed. The large hydration radius and the repulsive electrostatic forces likely hinder the adsorption of Ca^2+^. However, at pH above 13.3, the dominant species in solution is the Ca(OH)^+^ complex and not Ca^2+^.^55,56^ It is expected that hydroxide ions will act as an adsorption bridge for Ca^2+^, as observed on gibbsite^57^ and suggested by montmorillonite and kaolinite,^58^ as well as illite^59^ clays in aqueous calcium hydroxide solutions. The amount of adsorbed Ca^2+^ and OH^–^ suggests an extensive coverage on the basal surfaces of those clays.^58,59^ The faster uptake of ions by kaolinite in comparison to montmorillonite could be explained by the aluminate-terminated basal surface of kaolinite, which has a high hydroxyl surface density, while the montmorillonite basal surface is a flat silicate surface without out-of-plane oxygens.

The zeta potential is related to the charge state of the surface and gives clues regarding the physical and chemical properties of interfacial systems.^52^ Zeta potential values of C-S-H in high pH and calcium-rich solutions are consistently highly positive,^16,17,49,60^ suggesting an overcompensation of the negative surface charge by strongly adsorbed counterions, which are expected to be relatively immobile.^52^ Experimental zeta potentials on C-S-H with Ca/Si = 1.4 are negative when titrated with CaCl_2_ and positive when titrated with Ca(OH)_2_,^16^ indicating the role of hydroxide ions in the adsorption process. Further, in the synthesis of phase pure C-S-H, a correlation between pH and Ca/Si is observed. To achieve higher Ca/Si, higher pHs are needed.^9,56^ This again suggests an essential role of hydroxide ions in the precipitation of C-S-H.

**Figure 6:**
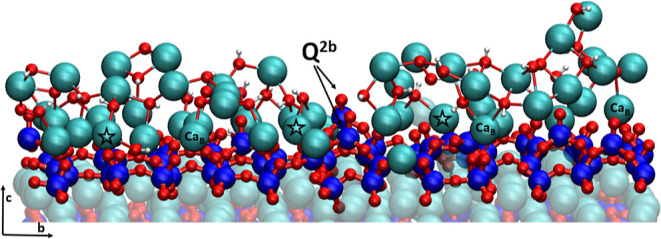
Calcium-hydroxyl network on the Mixed-Surface. Arrows point to deprotonated Q^2b^ silicates, Ca_B_ marks calcium in the bridging site, while stars mark inner sphere adsorbed calcium, which are the starting points for the Ca-OH network. For clarity, only selected calcium atoms are marked. Ca-OH connectivity is shown with “bonds”. Water molecules are not shown for clarity. Color code: Ca-cyan, Si-blue, O-red, and H-white.

The Mixed-Surface with additional calcium on the surface requires hydroxide ions to mediate the absorption of all the calcium in proximity of the surface. While it could be thought that Ca^2+^ and Ca(OH)^+^ would adsorb onto the deprotonated silanol groups of the Q^2b^ silicates, this adsorption behavior is rarely observed (Section S6). Roughly 30% of the solution Ca^2+^ is inner sphere adsorbed, exclusively on top of a dimer (Q^1^–Q^1^ or Q^2p^–Q^2p^). This adsorption site was previously reported by Kalinichev et al.^61^ While Ca^2+^ coordinates to at least two oxygens of the silicate dimer and sometimes simultaneously to the deprotonated silanol groups of the opposite silicate chain Q^2b^, a hydroxide is acting as an adsorption bridge to the main layer calcium. The MD results suggest the formation of a calcium-hydroxide network on top of the (001) surface, which starts from Ca_B_ and the inner sphere adsorbed calcium on top of the dimers (Figure 6, and Supporting Information 6).

### 3.5 Surface Charge

Labbez et al.^49^ carried out a combined experimental and theoretical approach in order to investigate the charging and electrokinetic behavior of C-S-H. While the model was very simplistic, it successfully predicted the experimentally obtained zeta potential. In a later study, Churakov et al.^62^ investigated the intrinsic acidity of surface sites in C-S-H with the use of density functional theory ab initio molecular dynamics, further confirming the results of Labbez et al.

The experiments were carried out on C-S-H with Ca/Si = 0.66, for which an Si-Surface 2 can be assumed (SSD = 4.8 OH/nm^2^). Labbez et al. used the model to predict the ionization fraction, α, and surface charge density, σ, for surface silanol densities of 2.8 and 0.8 in solution with 2 mM CaX_2_ (where X is a monovalent anion) at varying pH. The ionization fraction gives the percentage of deprotonated silanol groups and was used for our prediction of the surface charge density. Considering our newly proposed definition of SSD, one can back calculate the MCL of the assumed structures and predict the corresponding Ca/Si ratio of the studied C-S-H. The 2.8 and 0.8 SSDs correspond to C-S-H with Ca/Si ratios of 1.1 and 2.1, which are the lower and upper limits of a realistic C-S-H structure, therefore offering a valuable case to test our new atomistic model.

**Figure 7:**
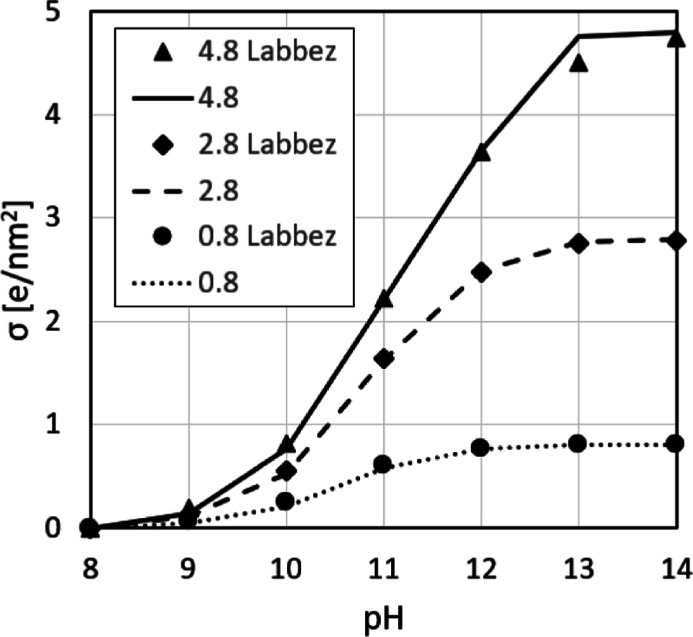
Surface charge density (σ) vs pH for different surface silanol densities (4.8, 2.8, and 0.8 OH/nm^2^) for our model (lines) and Grand Canonical Monte Carlo simulations from Labbez et al.^49^

The surface charge density (σ) calculation (Figure 7) follows the assumption that for each deprotonated silanol group, one calcium is adsorbed, effectively increasing the local charge by +1*e* per adsorbed calcium. At 100% deprotonation (pH 14),^49^ this results in a +2*e* charge per bridging silicate, resulting in a surface charge density equal to the surface silanol density. As can be seen from Figure 7, this is in very good agreement with the predicted surface charge density of Labbez et al.^49^ As explained previously, it is unlikely to adsorb so much Ca^2+^ onto the surface without the co-adsorption of hydroxide groups. However, the same surface charge can be achieved with the adsorption of *n*·Ca^2+^ and co-adsorption of 2(*n* – 2)·OH^–^ per deprotonated silanol group, where *n* is an integer. With this, the surface charge densities from Labbez et al. can be achieved while allowing for an additional amount of calcium at the surface, as suggested by experimental observations.^9^ Simultaneously, the calcium-hydroxide network on the surface increases the Ca-OH/Ca ratio of the C-S-H nanofoil. With this, an excellent agreement of the proposed C-S-H nanofoil model and experimentally determined C-S-H characteristics is achieved (Table 2).

**Table 2:** Comparison of C-S-H Characteristics of the C-S-H Nanofoil Model (Bulk, Surface, and Overall) with the Experimental Ones

property	C-S-H bulk	C-S-H surface	C-S-H nanofoil	experimental^3^
Ca/Si	1.72	1.66	1.7	1.7
MCL	3.0	3.0	3.0	3.0
Si-OH/Si	11%	2.1%^a^	9%	9%
Ca-OH/Ca	37%	44%	39%	45%

a 90% deprotonation of surface silanol groups assumed.

## 4 Discussion

The key assumptions of the C-S-H nanofoil model are as follows: (1) nanofoils are 3 layers, 2 interlayers thick; (2) the MCL of all the silicate chains in C-S-H is the same; and (3) the bulk Ca/Si ratio is similar to the overall nanofoil Ca/Si ratio, without considering the subsequent Ca^2+^ and OH^–^ adsorption on the surface, as discussed below. With these 3 assumptions, a C-S-H nanofoil model with the (001) basal surface for Ca/Si of 1.7 was constructed, which is in excellent agreement with experimentally measured C-S-H properties (Table 2). With the same assumptions, it is possible to construct C-S-H nanofoils with different Ca/Si ratios (see Section S1).

**Figure 8:**
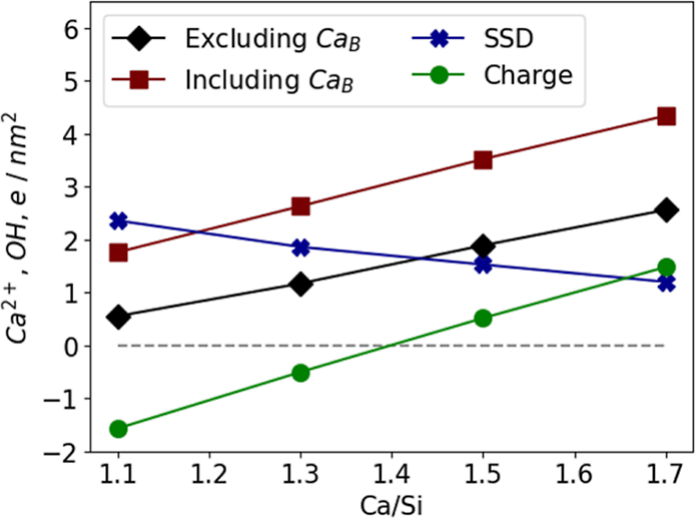
Surface silanol density (SSD in OH/nm^2^), Ca^2+^ adsorption (Ca^2+^/nm^2^), and surface charge density (e/nm^2^) at the (001) surface of the 3 layer, 2 interlayer thick C-S-H nanofoil as predicted by our model for different Ca/Si ratios. Including Ca_B_ (calcium in the bridging site of the silicate chains) accounts for all calcium at the surface. Excluding Ca_B_ accounts only for Ca^2+^, which is predicted to form the Ca^2+^-OH^–^ network and contributes to the surface charge. Ca_B_ fulfills the charge neutrality in its adsorption site and therefore does not contribute to the surface charge. Details on the model structures are listed in Section S1.

With this model, the adsorbed Ca^2+^ at the (001) surface (Ca^2+^/nm^2^) can then be modeled. As seen in Figure 8, with the increase in the Ca/Si ratio, an increase in Ca^2+^/nm^2^ is expected. This increased adsorption of Ca^2+^ agrees with the experimental observation of calcium concentrations in the supernatant of phase pure synthetic C-S-H as predicted by thermodynamic modeling and inductively coupled plasma (ICP) measurements.^9^ Thermodynamic modeling consistently predicts a higher amount of calcium in the supernatant than measured by ICP (3.8 mmol/L versus 0.39 mmol/L at Ca/Si = 1.7),^9^ and the Ca^2+^ adsorbed on the surface can explain this deficit.

The newly proposed surface model can explain some previously unclear observations about zeta potentials. Viallis-Terrisse et al.,^17^ Labbez et al.,^49^ and Yoshida et al.^16^ reported negative experimental values of zeta potential for C-S-H with Ca/Si of 0.66 and 1.34 in solutions with [Ca^2+^] < 2 mM. As suggested by our model, the surface charge for Ca/Si < 1.4 is expected to be negative (charge in Figure 8). A negative surface charge corresponds to negative zeta potentials, as explained below. When initial suspensions were titrated with Ca(OH)_2_, an increase in the zeta potential was observed with sign reversal at [Ca^2+^] ≈ 2 mM, suggesting further adsorption of Ca^2+^ and OH^–^ at the surface.

Labbez et al.^49^ increased the pH of the solution by addition of NaOH, whereby a decrease in the zeta potential was observed. This observation could be explained by OH^–^ adsorption or the replacement of Ca^2+^ by Na^+^ at the surface.^44,59^ Both mechanisms would result in lower surface charges and, therefore, in lower zeta potentials.

Haas and Nonat reported positive zeta potentials for samples with Ca/Si > 0.9 in solutions with [Ca^2+^] > 2 mM. This could be explained by an excess of positive charge at the surface due to Ca^2+^ and OH^–^ adsorption, as found for this model.^64^

The relationship between surface charge densities, Ca^2+^ and OH^–^ adsorption, and zeta potentials can be explained as follows: The proposed co-adsorption of Ca^2+^ and OH^–^ (see section Surface Charge) increases the surface charge by +1*e* per deprotonated surface silanol group. The SSD (OH/nm^2^), along with the predicted Ca^2+^/nm^2^, is shown in Figure 8. In Figure 8, the measure including Ca_B_ represents the total amount of calcium at the C-S-H (001) basal surface, which includes the calcium in the bridging site (Ca_B_) as well as Ca^2+^, which is part of the Ca^2+^-OH^–^ network. Excluding Ca_B_ accounts only for Ca^2+^, which forms the adsorbed network and effectively contributes to the charged state of the nanofoil. Experimental observations show that a pH above 12.5 is needed for C-S-H to have a nanofoil morphology.^56^ At such pHs, it is expected that more than 90% of the surface silanol groups will be deprotonated (see Labbez et al.^49^ and Table S11). From the predicted amount of adsorbed Ca^2+^ (and OH^–^) and the assumption of a 90% deprotonated (001) surface, one can calculate the expected surface charge at a given Ca/Si ratio. As seen in Figure 8, our C-S-H nanofoil model predicts negative surface charges below Ca/Si = 1.4 and positive surface charges for higher Ca/Si ratios.

**Figure 9:**
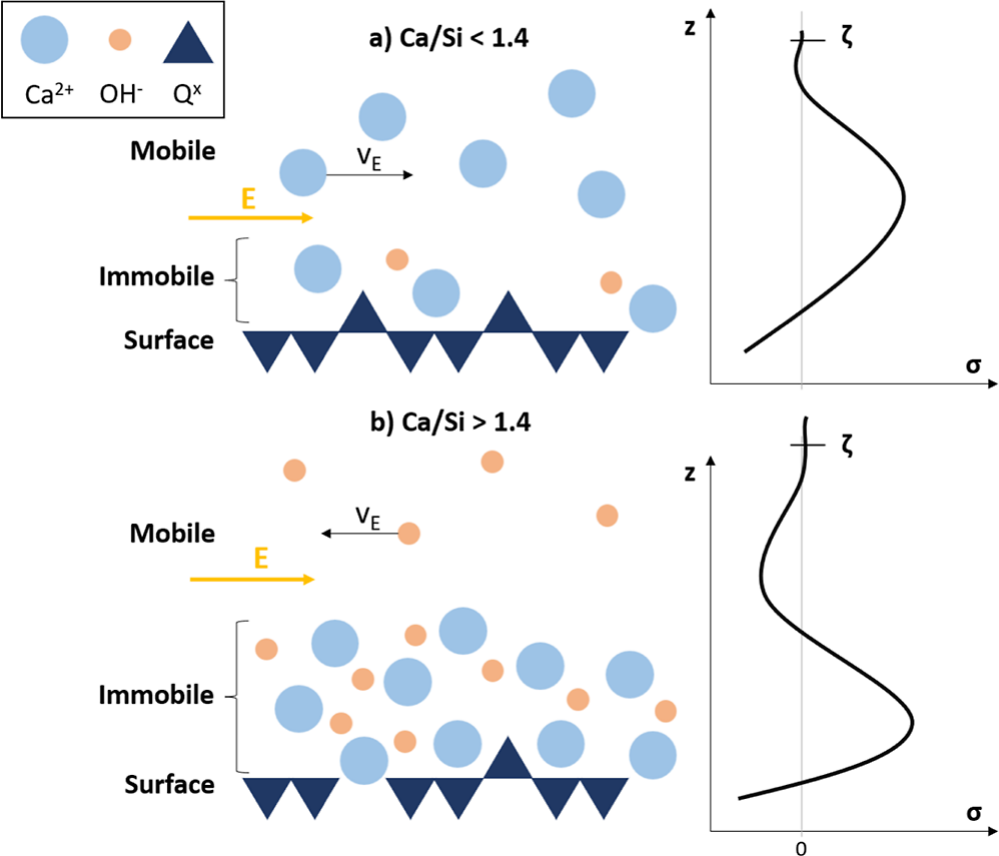
Schematic representation of the (001) C-S-H surface (a) for nanofoils with Ca/Si < 1.4, where a negative charge density (σ) is expected, and (b) for nanofoils with Ca/Si > 1.4, where a positive surface charge is expected. The zeta potential (ζ) is measured in the charge neutral region above the surface (*z*-axis direction), where charge neutrality and constant bulk mobilities are achieved. Q^*x*^ stands for silicate tetrahedra.

As explained by Předota et al.,^52,63^ the potential-driven velocities next to charged surfaces can be directly measured. Afterward, they can be divided by the applied field strength to provide electrophoretic or electro-osmotic mobilities, which can be converted to zeta potentials by various theoretical relations. Figure 9 shows the schematic of a C-S-H (001) surface of a nanofoil with a Ca/Si ratio below and above 1.4 and will be used to explain the expected zeta potential values.

As explained above, the charge density (σ) of the surface is negative due to the deprotonation of silanol groups on Q^2b^ silicates. A certain number of ions will be adsorbed on the surface (inner and outer sphere adsorption). These ions are expected to be rather immobile.^52^ As seen in Figure 8, the quantity of adsorbed Ca^2+^ (and OH^–^) for Ca/Si < 1.4 is expected to be less than needed to compensate for the negative surface charge. Therefore, the charge will be compensated by a region dominated by mobile cations above the surface (also referred to as the diffuse layer). The mobile ions of the diffuse layer determine the sign and amplitude of electrophoretic and electro-osmotic mobilities. In the case of mobile cations, the zeta potential is expected to be negative.^52,63^ For nanofoils with Ca/Si > 1.4, the surface is expected to have a high degree of Ca^2+^ (and OH^–^) adsorbed, resulting in positive charges in the immobile region. Therefore, the mobile region is expected to be anion dominated, which would result in positive zeta potentials.

Finally, after addressing the surface charge density of the nanofoil, some structural features of C-S-H can be addressed. Some experimental observations show a slow decrease in the Ca/Si ratio of C-S-H over time.^66^ By comparing our C-S-H nanofoil model with experimental data on zeta potential measurements, we conclude that the amount of Ca^2+^-OH^–^ adsorption depends on the solution condition (pH and [Ca^2+^]). Changes in pore solution (calcium concentration) over time most likely affect the adsorption and desorption of Ca^2+^ and OH^–^ and consequently cause a decrease in the Ca/Si ratio. However, the surface charge of C-S-H could remain unchanged, as theorized for clays in contact with lime solution.^59^ The comparison of pair distribution functions of C-S-H samples after 1 day and 1 year of hydration shows similar short-range ordering, suggesting that the bulk C-S-H structure remains unchanged.^65^ These findings further support the existence of the proposed Ca^2+^-OH^–^ adsorption network.

## 5 Conclusions

**Figure 10:**
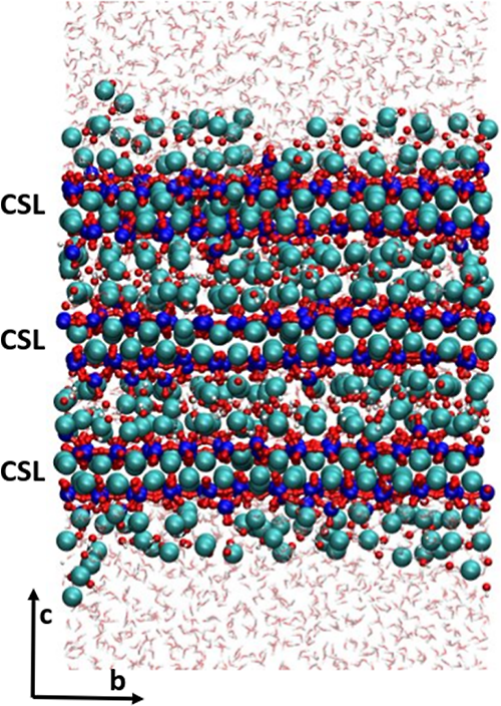
Snapshot of the full Ca/Si = 1.7 C-S-H nanofoil model with the mixed-surface termination and calcium-hydroxide adsorption, as described in Table 2. CSL—calcium-silicate layers. Color code: Ca-cyan, Si-blue, O-red, and H-white.

The present article shows an approach which considers multiple measurable characteristics of C-S-H for describing C-S-H nanofoils with the basal (001) surface. The characteristics which are used are the Ca/Si ratio, MCL (proportion of silicate species), and the relative concentration of silanol and hydroxide groups. The brick model^21^ was used to construct a model structure of the (001) C-S-H nanofoil with Ca/Si of 1.7, which best agrees with the collected experimental characteristics by Duque-Redondo et al.^3^ (Table 2 and Section S1.1) and is shown in Figure 10. There are four key findings:

- The comparison of the C-S-H nanofoil model with thickness and specific surface area measurements shows that the nanofoils consist of only 3 layers, 2 interlayers in thickness.
- The widely assumed Q^2b^-terminated silicate surface conflicts with the characteristics of C-S-H. A mixed silicate-calcium-terminated surface, with roughly 75% calcium termination at Ca/Si = 1.7, is in better agreement with the experimental data.
- The surface silanol density is a function of the MCL (and therefore the Ca/Si ratio).
- Calcium and hydroxide co-adsorption are needed to reach high Ca/Si ratios.

The C-S-H nanofoil model predicts the surface silanol density as a function of the MCL (and therefore the Ca/Si ratio) and the amount of adsorbed Ca^2+^ and OH^–^ at the surface as a function of the Ca/Si ratio (Figure 8). From these two parameters, the surface charge is calculated. The model predicts negative surface charges for Ca/Si < 1.4, which agrees with experimental zeta potential studies of C-S-H in solutions with [Ca^2+^] < 2 mM (Figure 9). The proposed calcium-hydroxide adsorption network provides an explanation for positively measured zeta potentials of C-S-H in contact with [Ca^2+^] > 2 mM solution.

With the use of the brick model,^21^ C-S-H surfaces which match the overall C-S-H characteristics can now be constructed and be used to help interpret experimental data and further investigate the interaction of other ions (e.g., chloride, sulfate, sodium) and molecules (accelerators, superplasticizers). This will lead to a far better understanding of surface phenomena that is of great importance for creating and using eco-friendly cements and concrete.

## Supporting Information Available

Details on model constriction, force field parameters, simulation protocol, and surface adsorption studies (PDF)
LAMMPS input files (ZIP)

## References

[ref1] Scrivener K. L.; John V. M.; Gartner E. M. Eco-Efficient Cements: Potential, Economically Viable Solutions for a Low-CO2, Cement-Based Materials Industry. Cem. Concr. Res. 2018, 114, 2–26. 10.1016/j.cemconres.2018.03.015.

[ref2] Heinz O.; Heinz H. Cement Interfaces: Current Understanding, Challenges, and Opportunities. Langmuir 2021, 37, 6347–6356. 10.1021/acs.langmuir.1c00617. 34000196

[ref3] Duque-Redondo E.; Bonnaud P. A.; Manzano H. A Comprehensive Review of C-S-H Empirical and Computational Models, Their Applications, and Practical Aspects. Cem. Concr. Res. 2022, 156, 106784 10.1016/j.cemconres.2022.106784.

[ref4] Ouzia A.; Scrivener K. The Needle Model: A New Model for the Main Hydration Peak of Alite. Cem. Concr. Res. 2019, 115, 339–360. 10.1016/j.cemconres.2018.08.005.

[ref5] Wilson W.; Gonthier J. N.; Georget F.; Scrivener K. L. Insights on Chemical and Physical Chloride Binding in Blended Cement Pastes. Cem. Concr. Res. 2022, 156, 106747 10.1016/j.cemconres.2022.106747.

[ref6] Bazzoni A.; Ma S.; Wang Q.; Shen X.; Cantoni M.; Scrivener K. L. The Effect of Magnesium and Zinc Ions on the Hydration Kinetics of C3S. J. Am. Ceram. Soc. 2014, 97, 3684–3693. 10.1111/jace.13156.

[ref7] Zhu X.; Richardson I. G. Morphology-Structural Change of C-A-S-H Gel in Blended Cements. Cem. Concr. Res. 2023, 168, 107156 10.1016/j.cemconres.2023.107156.

[ref8] Kumar A.; Walder B. J.; Kunhi Mohamed A.; Hofstetter A.; Srinivasan B.; Rossini A. J.; Scrivener K.; Emsley L.; Bowen P. The Atomic-Level Structure of Cementitious Calcium Silicate Hydrate. J. Phys. Chem. C 2017, 121, 17188–17196. 10.1021/acs.jpcc.7b02439.

[ref9] Harris M.; Simpson G.; Scrivener K.; Bowen P. A Method for the Reliable and Reproducible Precipitation of Phase Pure High Ca/Si Ratio (>1.5) Synthetic Calcium Silicate Hydrates (C-S-H). Cem. Concr. Res. 2022, 151, 106623 10.1016/j.cemconres.2021.106623.

[ref10] Andalibi M. R.; Kumar A.; Srinivasan B.; Bowen P.; Scrivener K.; Ludwig C.; Testino A. On the Mesoscale Mechanism of Synthetic Calcium-Silicate-Hydrate Precipitation: A Population Balance Modeling Approach. J. Mater. Chem. A 2018, 6, 363–373. 10.1039/c7ta08784e.

[ref11] Schönlein M.; Plank J. A TEM Study on the Very Early Crystallization of C-S-H in the Presence of Polycarboxylate Superplasticizers: Transformation from Initial C-S-H Globules to Nanofoils. Cem. Concr. Res. 2018, 106, 33–39. 10.1016/j.cemconres.2018.01.017.

[ref12] Tajuelo Rodriguez E.; Richardson I. G.; Black L.; Boehm-Courjault E.; Nonat A.; Skibsted J. Composition, Silicate Anion Structure and Morphology of Calcium Silicate Hydrates (C-S-H) Synthesised by Silica-Lime Reaction and by Controlled Hydration of Tricalcium Silicate (C3S). Adv. Appl. Ceram. 2015, 114, 362–371. 10.1179/1743676115Y.0000000038.

[ref13] Allen A. J.; Thomas J. J.; Jennings H. M. Composition and Density of Nanoscale Calcium-Silicate-Hydrate in Cement. Nat. Mater. 2007, 6, 311–316. 10.1038/nmat1871. 17384634

[ref14] Skinner L. B.; Chae S. R.; Benmore C. J.; Wenk H. R.; Monteiro P. J. M. Nanostructure of Calcium Silicate Hydrates in Cements. Phys. Rev. Lett. 2010, 104, 195502 10.1103/PhysRevLett.104.195502. 20866975

[ref15] Tennis P. D.; Jennings H. M. A model for two types of calcium silicate hydrate in the microstructure of Portland cement pastes. Cem. Concr. Res. 2000, 30, 855–863. 10.1016/S0008-8846(00)00257-X.

[ref16] Yoshida S.; Elakneswaran Y.; Nawa T. Electrostatic Properties of C–S–H and C-A-S-H for Predicting Calcium and Chloride Adsorption. Cem. Concr. Compos. 2021, 121, 104109 10.1016/j.cemconcomp.2021.104109.

[ref17] Viallis-Terrisse H.; Nonat A.; Petit J. C. Zeta-Potential Study of Calcium Silicate Hydrates Interacting with Alkaline Cations. J. Colloid Interface Sci. 2001, 244, 58–65. 10.1006/jcis.2001.7897.

[ref18] Jennings H. M. A model for the microstructure of calcium silicate hydrate in cement paste. Cem. Concr. Res. 2000, 30, 101–116. 10.1016/S0008-8846(99)00209-4.

[ref19] Brunauer S.; Kantro D. L.; Weise C. H. The Surface Energy of Tobermorite. Can. J. Chem. 1959, 37, 714–724. 10.1139/v59-097.

[ref20] Scrivener K.; Ouzia A.; Juilland P.; Kunhi Mohamed A. Advances in Understanding Cement Hydration Mechanisms. Cem. Concr. Res. 2019, 124, 105823 10.1016/j.cemconres.2019.105823.

[ref21] Kunhi Mohamed A.; Parker S. C.; Bowen P.; Galmarini S. An Atomistic Building Block Description of C-S-H - Towards a Realistic C-S-H Model. Cem. Concr. Res. 2018, 107, 221–235. 10.1016/j.cemconres.2018.01.007.

[ref22] Cong X.; Kirkpatrick R. 29Si MAS NMR Study of the Structure of Calcium Silicate Hydrate. Adv. Cem. Based Mater. 1996, 3, 144–156. 10.1016/s1065-7355(96)90046-2.

[ref23] Maddalena R.; Li K.; Chater P. A.; Michalik S.; Hamilton A. Direct Synthesis of a Solid Calcium-Silicate-Hydrate (C-S-H). Constr. Build. Mater. 2019, 223, 554–565. 10.1016/j.conbuildmat.2019.06.024.

[ref24] Bonaccorsi E.; Merlino S.; Kampf A. R. The Crystal Structure of Tobermorite 14 Å (Plombierite), a C-S-H Phase. J. Am. Ceram. Soc. 2005, 88, 505–512. 10.1111/j.1551-2916.2005.00116.x.

[ref25] Kunhi Mohamed A.; Moutzouri P.; Berruyer P.; Walder B. J.; Siramanont J.; Harris M.; Negroni M.; Galmarini S. C.; Parker S. C.; Scrivener K. L.; et al. The Atomic-Level Structure of Cementitious Calcium Aluminate Silicate Hydrate. J. Am. Chem. Soc. 2020, 142, 11060–11071. 10.1021/jacs.0c02988. 32406680

[ref26] Morales-Melgares A.; Casar Z.; Moutzouri P.; Venkatesh A.; Cordova M.; Kunhi Mohamed A.; Scrivener L.; Bowen P.; Emsley L. Atomic-Level Structure of Zinc-Modified Cementitious Calcium Silicate Hydrate. J. Am. Chem. Soc. 2022, 144, 22915–22924. 10.1021/jacs.2c06749. 36508687 PMC9782795

[ref27] Richardson I. G. Model Structures for C-(A)-S-H(I). Acta Crystallogr., Sect. B: Struct. Sci., Cryst. Eng. Mater. 2014, 70, 903–923. 10.1107/S2052520614021982. PMC4468512 25449614

[ref28] Hamid S. Α. The Crystal Structure of the 11Å Natural Tobermorite Ca2.25[Si3O7.5(OH)1.5].1H2O. Z. für Kristallogr.—Cryst. Mater. 1981, 154, 189–198. 10.1524/zkri.1981.154.3-4.189.

[ref29] Brunet F.; Bertani P.; Charpentier T.; Nonat A.; Virlet J. Application of 29Si Homonuclear and 1H-29Si Heteronuclear NMR Correlation to Structural Studies of Calcium Silicate Hydrates. J. Phys. Chem. B 2004, 108, 15494–15502. 10.1021/jp031174g.

[ref30] Kunhi Mohamed A.; Weckwerth S. A.; Mishra R. K.; Heinz H.; Flatt R. J. Molecular Modeling of Chemical Admixtures; Opportunities and Challenges. Cem. Concr. Res. 2022, 156, 106783 10.1016/j.cemconres.2022.106783.

[ref31] Jamil T.; Javadi A.; Heinz H. Mechanism of Molecular Interaction of Acrylate-Polyethylene Glycol Acrylate Copolymers with Calcium Silicate Hydrate Surfaces. Green Chem. 2020, 22, 1577–1593. 10.1039/c9gc03287h.

[ref32] Hou D.; Li T.; Wang P. Molecular Dynamics Study on the Structure and Dynamics of NaCl Solution Transport in the Nanometer Channel of CASH Gel. ACS Sustain. Chem. Eng. 2018, 6, 9498–9509. 10.1021/acssuschemeng.8b02126.

[ref33] Duque-Redondo E.; Kazuo Y.; López-Arbeloa I.; Manzano H. Cs-137 Immobilization in C-S-H Gel Nanopores. Phys. Chem. Chem. Phys. 2018, 20, 9289–9297. 10.1039/c8cp00654g. 29564427

[ref34] Androniuk I.; Landesman C.; Henocq P.; Kalinichev A. G. Adsorption of Gluconate and Uranyl on C-S-H Phases: Combination of Wet Chemistry Experiments and Molecular Dynamics Simulations for the Binary Systems. Phys. Chem. Earth 2017, 99, 194–203. 10.1016/j.pce.2017.05.005.

[ref35] Bu J.; Gonzalez Teresa R.; Brown K. G.; Sanchez F. Adsorption Mechanisms of Cesium at Calcium-Silicate-Hydrate Surfaces Using Molecular Dynamics Simulations. J. Nucl. Mater. 2019, 515, 35–51. 10.1016/j.jnucmat.2018.12.007.

[ref36] Honorio T.; Masara F.; Benboudjema F. Heat Capacity, Isothermal Compressibility, Isosteric Heat of Adsorption and Thermal Expansion of Water Confined in C-S-H. Cement 2021, 6, 100015 10.1016/j.cement.2021.100015.

[ref37] Svenum I. H.; Ringdalen I. G.; Bleken F. L.; Friis J.; Höche D.; Swang O. Structure, Hydration, and Chloride Ingress in C-S-H: Insight from DFT Calculations. Cem. Concr. Res. 2020, 129, 105965 10.1016/j.cemconres.2019.105965.

[ref38] Basquiroto de Souza F.; Sagoe-Crentsil K.; Duan W. Determining the Disordered Nanostructure of Calcium Silicate Hydrate (C-S-H) from Broad X-Ray Diffractograms. J. Am. Ceram. Soc. 2022, 105, 1491–1502. 10.1111/jace.18132.

[ref39] Abdolhosseini Qomi M. J.; Brochard L.; Honorio T.; Maruyama I.; Vandamme M. Advances in Atomistic Modeling and Understanding of Drying Shrinkage in Cementitious Materials. Cem. Concr. Res. 2021, 148, 106536 10.1016/j.cemconres.2021.106536.

[ref40] Thompson A. P.; Aktulga H. M.; Berger R.; Bolintineanu D. S.; Brown W. M.; Crozier P. S.; in ’t Veld P. J.; Kohlmeyer A.; Moore S. G.; Nguyen T. D.; et al. LAMMPS - a Flexible Simulation Tool for Particle-Based Materials Modeling at the Atomic, Meso, and Continuum Scales. Comput. Phys. Commun. 2022, 271, 108171 10.1016/j.cpc.2021.108171.

[ref41] Valavi M.; Casar Z.; Kunhi Mohamed A.; Bowen P.; Galmarini S. Molecular Dynamic Simulations of Cementitious Systems Using a Newly Developed Force Field Suite ERICA FF. Cem. Concr. Res. 2022, 154, 106712 10.1016/j.cemconres.2022.106712.

[ref42] Mitchell P. J.; Fincham D. Shell Model Simulations by Adiabatic Dynamics. J. Phys.: Condens. Matter 1993, 5, 1031–1038. 10.1088/0953-8984/5/8/006.

[ref43] Tilocca A.; de Leeuw N. H.; Cormack A. N. Shell-Model Molecular Dynamics Calculations of Modified Silicate Glasses. Phys. Rev. B: Condens. Matter Mater. Phys. 2006, 73, 104209 10.1103/PhysRevB.73.104209.

[ref44] Döpke M. F.; Lützenkirchen J.; Moultos O. A.; Siboulet B.; Dufrêche J.-F.; Padding J. T.; Hartkamp R. Preferential Adsorption in Mixed Electrolytes Confined by Charged Amorphous Silica. J. Phys. Chem. C 2019, 123, 16711–16720. 10.1021/acs.jpcc.9b02975.

[ref45] Cygan R. T.; Liang J. J.; Kalinichev A. G. Molecular Models of Hydroxide, Oxyhydroxide, and Clay Phases and the Development of a General Force Field. J. Phys. Chem. B 2004, 108, 1255–1266. 10.1021/jp0363287.

[ref46] Shahsavari R.; Pellenq R. J. M.; Ulm F. J. Empirical Force Fields for Complex Hydrated Calcio-Silicate Layered Materials. Phys. Chem. Chem. Phys. 2011, 13, 1002–1011. 10.1039/c0cp00516a. 21069228

[ref47] Müser M. H.; Sukhomlinov S. V.; Pastewka L. Interatomic Potentials: Achievements and Challenges. Adv. Phys.: X 2023, 8, 2093129 10.1080/23746149.2022.2093129.

[ref48] Mamatkulov S.; Fyta M.; Netz R. R. Force Fields for Divalent Cations Based on Single-Ion and Ion-Pair Properties. J. Chem. Phys. 2013, 138, 024505 10.1063/1.4772808. 23320702

[ref49] Labbez C.; Jönsson B.; Pochard I.; Nonat A.; Cabane B. Surface Charge Density and Electrokinetic Potential of Highly Charged Minerals: Experiments and Monte Carlo Simulations on Calcium Silicate Hydrate. J. Phys. Chem. B 2006, 110, 9219–9230. 10.1021/jp057096+. 16671737

[ref50] Sanchez F.; Zhang L. Molecular Dynamics Modeling of the Interface between Surface Functionalized Graphitic Structures and Calcium-Silicate-Hydrate: Interaction Energies, Structure, and Dynamics. J. Colloid Interface Sci. 2008, 323, 349–358. 10.1016/j.jcis.2008.04.023. 18486142

[ref51] Labbez C.; Nonat A.; Pochard I.; Jönsson B. Experimental and Theoretical Evidence of Overcharging of Calcium Silicate Hydrate. J. Colloid Interface Sci. 2007, 309, 303–307. 10.1016/j.jcis.2007.02.048. 17346727

[ref52] Předota M.; Machesky M. L.; Wesolowski D. J. Molecular Origins of the Zeta Potential. Langmuir 2016, 32, 10189–10198. 10.1021/acs.langmuir.6b02493. 27643625

[ref53] Janusz W.; Patkowski J.; Chibowski S. Competitive Adsorption of Ca2+ and Zn(II) Ions at Monodispersed SiO2/Electrolyte Solution Interface. J. Colloid Interface Sci. 2003, 266, 259–268. 10.1016/S0021-9797(03)00469-7. 14527448

[ref54] Bischoff M.; Biriukov D.; Předota M.; Marchioro A. Second Harmonic Scattering Reveals Ion-Specific Effects at the SiO2 and TiO2 Nanoparticle/Aqueous Interface. J. Phys. Chem. C 2021, 125, 25261–25274. 10.1021/acs.jpcc.1c07191. PMC9109693 35591899

[ref55] Kulik D.; Berner U.; Curti E. Modelling Chemical Equilibrium Partitioning with the GEMS-PSI Code; Paul Scherrer Institut: Switzerland, 2004, Technical report (CH-0401); pp 109–123.

[ref56] Shen X.; Feng P.; Zhang Q.; Lu J.; Liu X.; Ma Y.; Jin P.; Wang W.; Ran Q.; Hong J. Toward the Formation Mechanism of Synthetic Calcium Silicate Hydrate (C-S-H) - PH and Kinetic Considerations. Cem. Concr. Res. 2023, 172, 107248 10.1016/j.cemconres.2023.107248.

[ref57] Siretanu I.; Ebeling D.; Andersson M. P.; Stipp S. L. S.; Philipse A.; Stuart M. C.; Van Den Ende D.; Mugele F. Direct Observation of Ionic Structure at Solid-Liquid Interfaces: A Deep Look into the Stern Layer. Sci. Rep. 2014, 4, 4956 10.1038/srep04956. 24850566 PMC4030399

[ref58] Diamond S.; Kinter E. B. Adsorption of Calcium Hydroxide by Montmorillonite and Kaolinite. J. Colloid Interface Sci. 1966, 22, 240–249. 10.1016/0021-9797(66)90029-4.

[ref59] Konan K. L.; Peyratout C.; Bonnet J. P.; Smith A.; Jacquet A.; Magnoux P.; Ayrault P. Surface Properties of Kaolin and Illite Suspensions in Concentrated Calcium Hydroxide Medium. J. Colloid Interface Sci. 2007, 307, 101–108. 10.1016/j.jcis.2006.10.085. 17174321

[ref60] Nägele E. The Zeta-Potential of Cement. Cem. Concr. Res. 1985, 15, 453–462. 10.1016/0008-8846(85)90118-8.

[ref61] Kalinichev A. G.; Wang J.; Kirkpatrick R. J. Molecular Dynamics Modeling of the Structure, Dynamics and Energetics of Mineral–Water Interfaces: Application to Cement Materials. Cem. Concr. Res. 2007, 37, 337–347. 10.1016/j.cemconres.2006.07.004.

[ref62] Churakov S. V.; Labbez C.; Pegado L.; Sulpizi M. Intrinsic Acidity of Surface Sites in Calcium Silicate Hydrates and Its Implication to Their Electrokinetic Properties. J. Phys. Chem. C 2014, 118, 11752–11762. 10.1021/jp502514a.

[ref63] Biriukov D.; Fibich P.; Předota M. Zeta Potential Determination from Molecular Simulations. J. Phys. Chem. C 2020, 124, 3159–3170. 10.1021/acs.jpcc.9b11371.

[ref64] Haas J.; Nonat A. From C–S–H to C–A–S–H: Experimental Study and Thermodynamic Modelling. Cem. Concr. Res. 2015, 68, 124–138. 10.1016/j.cemconres.2014.10.020.

[ref65] Grangeon S.; Fernandez-Martinez A.; Baronnet A.; Marty N.; Poulain A.; Elkaïm E.; Roosz C.; Gaboreau S.; Henocq P.; Claret F. Quantitative X-Ray Pair Distribution Function Analysis of Nanocrystalline Calcium Silicate Hydrates: A Contribution to the Understanding of Cement Chemistry. J. Appl. Crystallogr. 2017, 50, 14–21. 10.1107/S1600576716017404. 28190991 PMC5294392

[ref66] Zunino F.; Scrivener K. Microstructural Developments of Limestone Calcined Clay Cement (LC3) Pastes after Long-Term (3 Years) Hydration. Cem. Concr. Res. 2022, 153, 106693 10.1016/j.cemconres.2021.106693.

