# Supplementary material for: Atomic-Level and Surface Structure of Calcium Silicate Hydrate Nanofoils
Source: J Phys Chem C Nanomater Interfaces. 2023 Sep 8;127(37):18652–61. doi: 10.1021/acs.jpcc.3c03350 (PMC10518866; doi:10.1021/acs.jpcc.3c03350)
Supplement: Supplementary file 1 — jp3c03350_si_001.pdf [file jp3c03350_si_001.pdf]

Supporting Information

Atomic-Level and Surface Structure of Calcium Silicate Hydrate Nanofoils

Ziga Casar[1+], Aslam Kunhi Mohamed[2*], Paul Bowen[1], Karen Scrivener[1]

[1] Laboratory of Construction Materials, Institut des Matériaux, Ecole Polytechnique Fédérale de Lausanne (EPFL), CH-1015 Lausanne, Switzerland

[2] Institute for building Materials, Department of Civil, Environmental and Geomatic Engineering, ETH Zürich, CH-8093 Zürich, Switzerland

[*] Current address: Department of Civil Engineering, IIT Madras, Chennai-600036, Tamil Nadu, India

[+] Corresponding author's email: ziga.casar@epfl.ch

Table of Content

1 SURFACE CONSTRUCTION 2

1.1 EXPERIMENTAL DATA 3

1.2 C-S-H NANOFOIL WITH MIXED-SURFACE 3

1.3 COMPARISON OF DIFFERENT THICKNESSES 9

2 FORCE FIELD 9

3 SIMULATION PROTOCOL 11

4 SURFACE CHARGE DENSITY 12

5 CALCIUM ADSORPTION: SI-SURFACE 2 13

6 CALCIUM-HYDROXIDE SURFACE NETWORK 15

REFERENCES 19

1 Surface construction

The Ca-Si chains for the surface are composed of the same groups of atoms (figure S1) as the upper and lower Ca-Si chains of the main layer.[1] Some of the different combinations of atom groups that can be used to form a single surface block are shown in figure S1a. Surface blocks 1 to 4 give a silicon termination of the C-S-H surface, where 1 and 2 show a block with the missing Q^{2b} site, and 5 to 8 give calcium termination, where we have a calcium in the bridging site (5 and 6), or calcium in the bridging site with an additional calcium next to it (7 and 8). The surface charge arises from the pH-dependent protonation of surface oxygens and deprotonation of chemisorbed water molecules[2], which can be accounted for in the model as seen in pairs 1-2, 3-4 and 5-6, leading to a net charge. Although not shown in figure S1a, partial protonation and deprotonation, or additional hydroxides in the case of calcium termination are possible. Since classical molecular dynamics does not account for bond breakage and creation, the degree of silanol deprotonation as well as hydroxide presence on the surface needs to be chosen beforehand. Figure S1b shows an assembly of different surface blocks to form a mixed Ca-Si surface chain in the b-axis direction.

a)

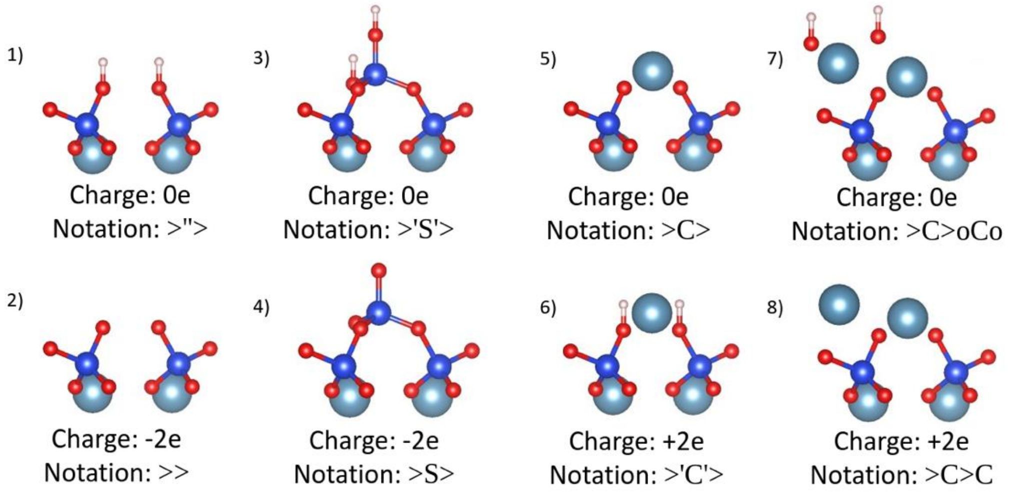

b)

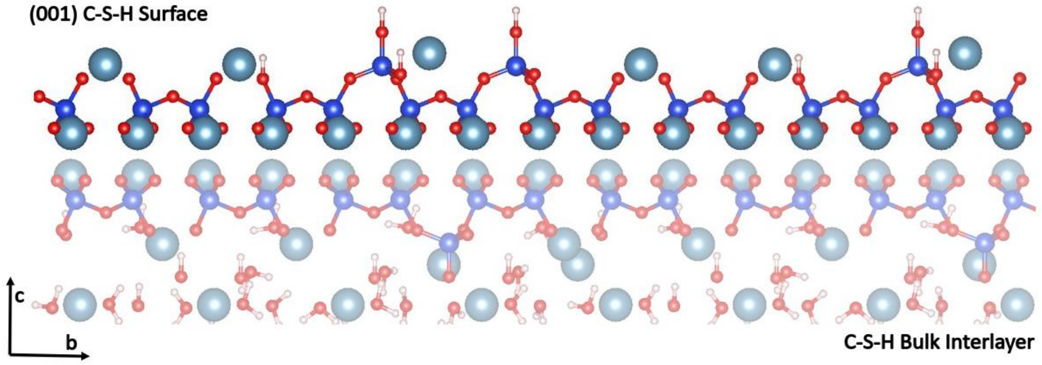

Figure S1. a) Possible C-S-H surface blocks, with net charge and building block notation[1] for the upper surface (in c direction); b) Mixed Ca-Si surface chain constructed from different surface blocks, (for the upper surface). Color code: Turquoise – calcium, dark blue – silicon, red – oxygen, white – hydrogen.

As is the case for the C-S-H bulk structure,[1] there can be many possible local arrangements of different surface blocks to build a representative C-S-H surface. Local arrangements here refer to the length of individual silicate chains (dimer, pentamer, …), the protonation of silanol groups and location of calcium and hydroxide ions at the surface. It is crucial that the constructed C-S-H structure (bulk with surface) mimics the experimental results as accurately as possible.[3] The Ca/Si ratio significantly affects a wide range of phenomena.[3–10] In particular the water structure at the surface will be directly related to the surface termination, and this will determine the hydrodynamic properties of flows through cement porosity,[3,11] since it can be substantially different to bulk water.[12]

1.1 Experimental data

Figure S2 shows the collected experimental data by Duque-Redondo et al.[13] which was used throughout the manuscript. The MCL values are from references [14–18], while the Si-OH/Si and Ca-OH/Ca values are from [19–22].

The round colored markers show the data points which were used to construct the atomic-scale model of the C-S-H nanofoils. The exact values of the markers are given in table S1.

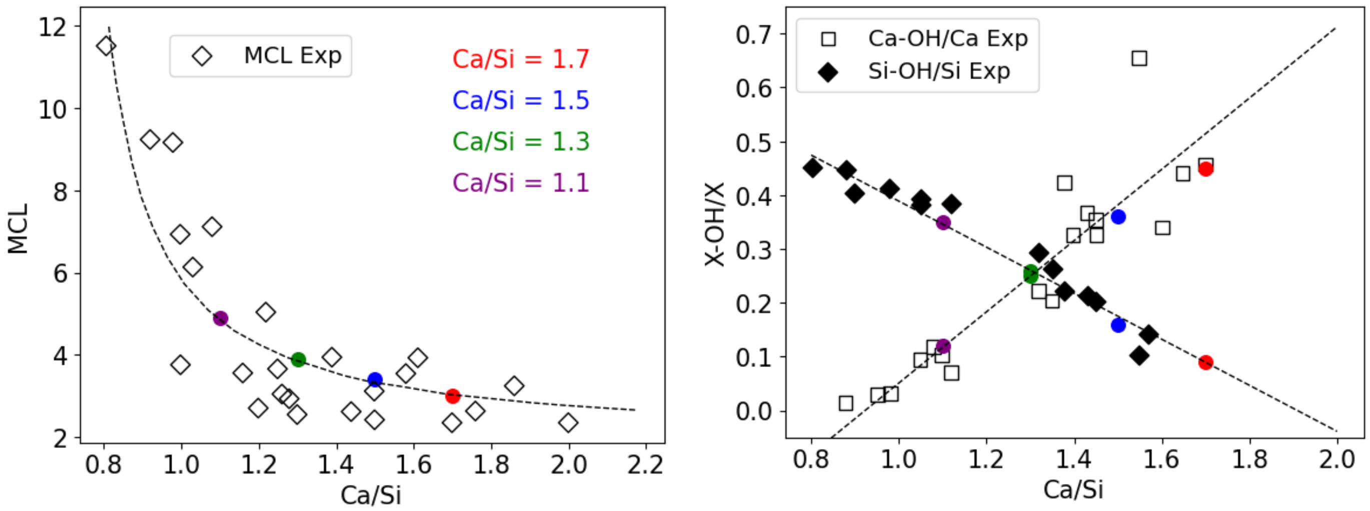

Figure S2. Collected experimental data by Duque-Redondo et al.[13] Colors correspond to the values which were used through the manuscript to construct the atomic-scale models of C-S-H nanofoils at a given Ca/Si and thickness.

Table S1. Values which were taken for the construction of the C-S-H nanofoils through the manuscript. The values were chosen from the collected data by Duque-Redondo et al.[13] and are highlighted in figure S2.

Ca/Si	MCL	Si-OH/Si	Ca-OH/Ca
1.1	4.9	0.35	0.12
1.3	3.9	0.25	0.25
1.5	3.4	0.16	0.36
1.7	3.0	0.09	0.45

1.2 C-S-H nanofoil with Mixed-Surface

This section provides a detailed explanation on how to construct a C-S-H nanofoil model with the (001) Mixed-Surface termination for any given Ca/Si ratio.

First the size of the system model needs to be specified:

- A = number of defective tobermorite 14Å unit cells in a-axis direction
- B = number of defective tobermorite 14Å unit cells in b-axis direction
- L = number of calcium-silicate sheets (layers) in z-axis direction ($L = 3$ for 3 layers, 2 interlayers C-S-H)

The mean chain length (MCL) is defined[1,23] as

$$MCL = \frac{Q^i}{0.5 \cdot Q^{i \neq 2b} - Q^{2b}} \tag{1}$$

where Q^i denotes either the exact number of given Q species in the atomistic model, or the proportion of Q species as given by ^{29}Si NMR.[23] i is either $2b$, $2p$ or 1. Each Q^{2b} is always accompanied by two Q^{2p} silicates, therefore the MCL can be also written as

$$MCL = \frac{Q^1 + 3Q^{2b}}{0.5 \cdot Q^1} \tag{2}$$

The maximum amount of Q species (Q^{MAX}) for a given model of the nanofoil equals to

$$Q^{MAX} = Q^1 + Q^{2b} + Q^{2p} + Ca_B \tag{3}$$

where Ca_B stands for all the bridging sites, which are missing a Q^{2b} silicate, and are presumably occupied with a calcium atom. Due to the structure of the silicate chains, one third of the Q^{MAX} are linking the dimers. This gives the next relation:

$$\frac{Q^{MAX}}{3} = Q^{2b} + Ca_B \tag{4}$$

Combining equations (2), (3) and (4) gives the number of Q^{2b} silicates, which is needed for ensuring the right MCL of the computational model (equation 5). The number of Q^{2b} silicates should be equally distributed between the calcium-silicate chains of the nanofoil.

$$Q^{2b} = \frac{2}{3}Q^{MAX}\frac{MCL - 2}{2 \cdot MCL + 2} \quad (5)$$

where Q^{MAX} is calculated from the parameters which decide the size of the atomistic system:

$$Q^{MAX} = 6 \cdot A \cdot B \cdot L \quad (6)$$

From the total amount of silicates (Si) in the system:

$$Si = \frac{2}{3}Q^{MAX} + Q^{2b} \quad (7)$$

the needed amount of calcium (Ca) for a targeted Ca/Si ratio can be calculated as

$$Ca = \left(\frac{Ca}{Si}\right)_{target} \cdot Si \quad (8)$$

Assuming there is no calcium missing from the main layer calcium-silicate sheets, one can calculate the amount of calcium atoms which is in the interlayers and on the surfaces, while excluding the amount of calcium in the bridging sites (Ca_B):

$$Ca_{int+surf} = Ca - \frac{2}{3}Q^{MAX} - Ca_B \quad (9)$$

where:

$$Ca_B = \frac{Q^{MAX}}{3} - Q^{2b} \quad (10)$$

As seen in the review paper of Duque-Redondo et al.[13] and figure S2 the Si-OH/Si ratio decreases linearly with the increase in Ca/Si ratio. The linear fit of the experimental results (see figure S2) yields:

$$\frac{Si - OH}{Si} = -0.4306\left(\frac{Ca}{Si}\right)_{target} + 0.8217 \quad (11)$$

Assuming a deprotonation rate of surface silanol groups (D_{surf} = 0.9 for 90% deprotonation), the number of silanol groups in the interlayers can be calculated as:

$$N_{Si-OH}^{Int} = \left(\frac{Si-OH}{Si}\right)_{eq11} \cdot Si - \frac{4}{2 \cdot L - 2}\left(1 - D_{surf}\right) \cdot Q^{2b} \tag{12}$$

Finally, the amount of calcium which is associated with the interlayer and surface (equation 9), needs to be divided between them. The packing of the interlayers with calcium will depend on the desired Ca/Si ratio of the bulk (Ca/Si_{bulk}):

$$Ca_{int} = \left(\frac{Ca}{Si}\right)_{bulk} \cdot Si_{bulk} - (2 \cdot L - 2) \cdot \left(2 \cdot A \cdot B + \frac{Ca_B}{2 \cdot L}\right) \tag{13}$$

$$Si_{bulk} = \frac{2 \cdot L - 2}{2 \cdot L} \cdot Si \tag{14}$$

$$Ca_{surf} = Ca_{int+surf} - Ca_{int} \tag{15}$$

Summary on constructing a C-S-H nanofoil with the (001) surfaces and targeted Ca/Si ratio

The C-S-H nanofoil was constructed with the in-house brick model code from Kunhi Mohamed et al.[1] (the code can be requested by contacting the authors). The example of the code is shown below.

Step-by-step guide to construct the nanofoil.

1. Define the targeted Ca/Si ratio and the size of the bulk C-S-H (number of layers – L, number of bricks in x-axis and y-axis direction – A and B). Use the Ca/Si ratio to define the MCL (figure S2). The MCL can also be calculated from the proportion of Q species (equation 2).

2. Calculate the nanofoil properties (Si, Q^{2b}, Ca, Ca_B, Ca_{Int}, Ca_{Surf}, …) with equations 4 to 15.

3. If starting from a CIF file of tobermorite 14 Å:

    1. Multiply the tobermorite unit cell in the x, y and z-axis direction to create a super cell of L+2 thickness, A number of unit cells in x-axis direction and B number of unit cells in y-axis direction.

    2. Delete the calcium silicate chains and interlayer Ca^{2+} and H_2O placed at z_{min} and z_{max}. With this a tobermorite nanofoil was constructed which has L layers and is terminated by (001) surfaces.

3. On each side of each layer remove $Ca_B/(2L)$ number of Q^{2b} silicates and replace them with Ca^{2+} ions. The Q^{2b} silicates are removed by removing the Si atom and two O atoms, which are not shared with the neighboring silicates. Remaining Q^{2b} silicates should be equally distributed on the individual layers.

4. On the surface deprotonate D_{surf} silanol groups. Start by deprotonating the upper silanol groups, followed by the lower (closer to the calcium oxide sheet) silanol groups. Silanol groups should be equally distributed on the surface.

5. On each interlayer surface distribute $N_{SiOH}^{Int}/(L-1/L)$ silanol groups. The silanol groups should be attached to the non-sharing oxygens of the Q^{2b} silicates. Silanol groups should be equally distributed throughout the interlayer, firstly placed on the silicate oxygens which are closer to the calcium oxide sheet.

6. The tobermorite interlayer holds one Ca^{2+} per brick. Add or remove so many Ca^{2+} in the interlayers that the total amount of Ca^{2+} (excluding Ca_B in the bridging sites) equals the calculated Ca_{Int}. Make sure each interlayer holds the same amount of Ca^{2+} and that they are equally distributed through it.

7. Calculate the charge of each interlayer. By removing one hydrogen from the water molecule, you create an OH^- ion which lowers the charge in the interlayer by -1 e. Create so many OH^- ions to achieve charge neutrality of the bulk. Distribute the OH^- equally through the interlayers.

8. If needed add additional H_2O molecules to the interlayers to raise the 2H/Si ratio. Make sure to equally distribute the H_2O molecules.

9. Close to each surface equally distribute $Ca_{surf}/2$ number of Ca^{2+}. Between the placed Ca^{2+} add OH^- ions. For charge neutral surface Ca_{surf} number of OH^- should be placed. If a positive surface is desired, place less OH^- ions, so many to obtain the desired surface charge.

10. Place a water slab above each surface.

4. If using the in-house brick model code:

    1. Follow the same steps (1 to 8) as in step 3 to generate a description of the C-S-H nanofoil according to the brick model nomenclature (example below). Use this code to generate the model structure.

    2. The brick code generates structures in DL Poly format which can be converted to XYZ format. This can be done for example with the visualization program VESTA.[24]

3. Packmol[25] (uses XYZ data format) can then be used to insert Ca_{surf}/2 number of Ca^{2+} and the desired number of OH^- ions in the proximity of each surface. Packmol can be also used to insert a water slab between the surfaces.

Example of the Brick Model[1] code of a C-S-H nanofoil measuring 3 layers, 2 interlayers in the c-axis direction, 5 bricks in the x-axis direction and 6 bricks in the y-axis direction, without Ca_{Surf} on the surface. For a detailed description of the brick model we refer the reader to the publication of Kunhi Mohamed et al.[1]

(001) surface

```
>C> >S'> >C> >C> >C> >C>
>C> >C> >S> >C> >S> >S>
>C> >C> >C> >C> >C> >C>
>C> >C> >S'> >C> >S'> >S>
>C> >C> >C> >C> >C> >C>
Z
```

Bulk (1 interlayer)

```
<S'<oX6>Co> <Co<oX6>oC> <S'<oX6>Co> <C<oCoX5>S> <Co<X6>oC> <S<X6>Co>
<oC<Xo6>Co> <C<oCoX5>oC> <Co<X6>oC> <C<X6>oC> <C<X6>S> <Co<Xo6>S'>
<S'<X6>S> <Co<X6>S'> <Co<oXC5>oC> <S<X6>oC> <S<XCo5>oC> <Co<X6>oC>
<oC<X6>oC> <S'<XC5>oC> <Co<X6>oC> <oC<X6>oC> <oC<oX6>C> <oC<X6>oC>
<oC<XC5>S> <Co<X6>S> <Co<X6>C> <Co<X6>oC> <S<Xo6>C> <Co<XCo5>oC>
Z
```

Bulk (1 interlayer)

```
<S'<oX6>Co> <Co<oX6>oC> <S'<oX6>Co> <C<oCoX5>S> <Co<X6>oC> <S<X6>Co>
<oC<Xo6>Co> <C<oCoX5>oC> <Co<X6>oC> <C<X6>oC> <C<X6>S> <Co<Xo6>S'>
<S'<X6>S> <Co<X6>S'> <Co<oXC5>oC> <S<X6>oC> <S<XCo5>oC> <Co<X6>oC>
<oC<X6>oC> <S'<XC5>oC> <Co<X6>oC> <oC<X6>oC> <oC<oX6>C> <oC<X6>oC>
<oC<XC5>S> <Co<X6>S> <Co<X6>C> <Co<X6>oC> <S<Xo6>C> <Co<XCo5>oC>
Z
```

(001) surface

```
<C< <S'< <C< <C< <C< <C<
<S< <C< <S< <C< <S'< <C<
<C< <C< <C< <C< <C< <C<
<C< <S'< <C< <C< <C< <C<
<S< <C< <C< <C< <C< <S<
```

1.3 Comparison of different thicknesses

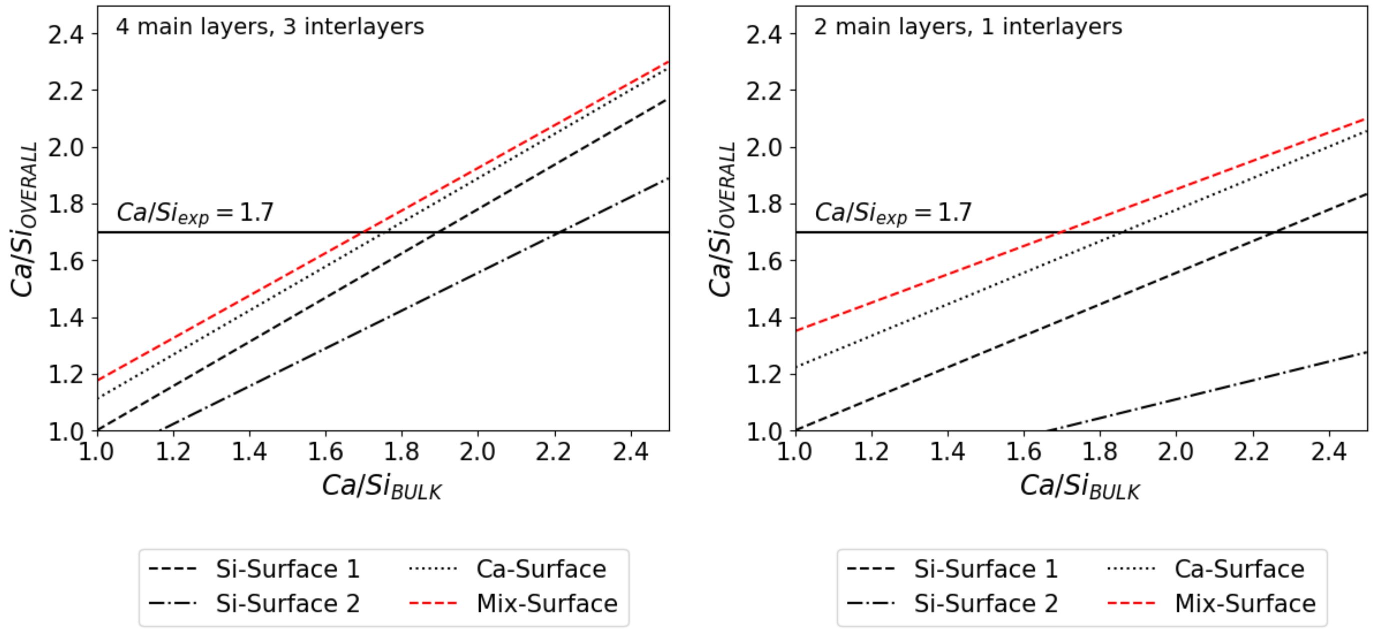

Figure S3. Correlation of the overall Ca/Si ratio on the Ca/Si of the bulk structure for the 4 layers, 3 interlayers and 2 layers, 1 interlayer C-S-H structures with given (001) surfaces terminations.

Table S2. Mean chain Length (MCL) and Ca/Si of investigated C-S-H nanofoils with overall Ca/Si = 1.7 and different surface terminations.

	MCL_{BULK}	$MCL_{SURFACE}$	Ca/Si_{BULK}
**4 layers, 3 interlayers**			
Si-Surface 1	3.5	2.0	1.9
Si-Surface 2	2.0	∞	2.2
Ca-Surface	3.5	2.0	1.76
Mix-Surface	3	3	1.7
**2 layers, 1 interlayer**			
Si-Surface 1	5.0	2.0	2.26
Si-Surface 2	1.2	∞	3.77
Ca-Surface	5.0	2.0	1.86
Mix-Surface	3	3	1.7

2 Force Field

In the following the force field parameters are listed. The force field is an improved version of the previous full charge force field of Valavi et al.[26] and Kunhi Mohamed et al.[27,28] The TIP4P/2005 water model[29] is used in conjunction with the SHAKE algorithm. All simulations were carried out with LAMMPS.[30] For the description of the used interatomic potentials we refer the reader to the LAMMPS documentation [30] or the SI section of Valavi et al. [26] The kspace solver used was the pppm/tip4p with an accuracy of 1e-4.

Table S3. Atomic charges.

Atomic species	Charge	Description
Ca	+2.0	Calcium
Si	+4.0	Silicon
O	+0.848	Silicate oxygen - core
O(S)	-2.848	Silicate oxygen - shell
Ow	-1.1128	Water oxygen
Hw	+0.5564	Water hydrogen
Oh	-1.4	Hydroxide oxygen
H	+0.4	Hydroxide hydrogen
Cl	-1.0	Chlorine

Table S4. Buckingham interatomic potentials.

	A [eV]	ρ [Å]	C [eV Å^6]
Ca-O(S)	2152.3566	0.309227	0.09944
Si-O(S)	1283.9	0.3205	10.66
Si-Ow	1283.556	0.3202	10.66
Si-Oh	983.5	0.3255	10.66
O(S)-O(S)	22764.3	0.149	27.88
O(S)-Ow	22764.3	0.149	28.92
O(S)-Oh	22764.3	0.149	13.94
O(S)-Hw	512.0	0.25	0.0

Table S5. Lennard-Jones interatomic potentials.

	ε [eV]	σ [Å]
Ca-Ca	0.0121188	2.8331
Ca-Ow	0.00984634	2.996
Ca-Oh	0.0151386	3.0613
Ca-Cl	0.00247555	3.87545
O(S)-Cl	0.00309242	4.103625
Ow-Ow	0.008	3.1589
Ow-Oh	0.012299878	3.28945
Ow-Cl	0.002113497	4.03835
Oh-Oh	0.018910874	3.42
Oh-Cl	0.00309242	4.1689
Cl-Cl	0.000505691	4.9178

Table S6. N-M interatomic potentials.

	E_0 [eV]	r_0 [Å]	n	m
O(S)-H	0.0073	2.71	9	6
Ow-H	0.055	2	9	6
Oh-H	0.0073	2.71	9	6

Table S7. Harmonic bonds.

	K [eV/Å^2]	r_0 [Å]
O-O(S)	37.5	0
Ow-Hw	9.7563	0.9572

Table S8. Morse bond.

	D [eV]	α [1/ Å]	r_0 [Å]
Oh-H	7.0525	3.1749	0.942

Table S9. Harmonic angles.

	K [eV]	ϴ [°]
O-Si-O<br>Oh-Si-O<br>Oh-Si-Oh	7.74815	109.47
Si-Oh-H	7.74815	141.5

3 Simulation protocol

Since the brick model inserts atoms in a predetermined way, which can result in atoms being in close proximity to each other, therefore experiencing large initial forces, a suitable equilibration protocol was used. CS ensemble refers to the core-shell equilibration. For the details on the core-shell equilibration we refer the reader to the work of Valavi et al.[26] The used thermostat is the Nose-Hoover with Tdamp values 100 times the timestep, while Pdamp was 1000 times the timestep.

Table S10. Simulation protocol.

Run	Timestep [ps]	Number of Timesteps	Ensemble	Atomic species	Temperature [K]	Pressure [bar]
1	0.0001	10e3	NVT	Ca,Ow,Hw	10 -> 300	
2	0.0001	100e3	NVT	all	10 -> 300	
3	0.00028	50e3	NVT	all	300	
4	0.00028	10e3	CS	all	300	
5	0.00028	960e3	NPT	all	300	1 bar (z direction)
6	0.00028	500e3	NPT	all	300	1 bar (x,y directions)
7	0.00028	60e3	NPT	all	300	1 bar (z direction)
8	0.00015	200e3	NVT	all	300 -> 700	
9	0.00015	200e3	NVT	all	700	
10	0.00015	100e3	NVT	all	700 -> 300	
11	0.00015	60e3	NPT	all	300	1 bar (z direction)
12	0.00028	70e6	NVT	all	300	

In the first run only calcium and water are thermostated. This is done with a smaller timestep so that the calcium and water can move away from the hydroxides (the code packs them relatively close). A slow equilibration follows, where the entire system is heated to the desired temperature. Next (4) is the core-shell equilibration, where the core-shell movement is decoupled and rescaled, which is necessary to prevent the overheating of core-shell pairs and keeping the total energy of the system constant.[31] From (5) to (7) a series of NPT runs is carried out in order to relax the simulation box in the z-direction (obtaining bulk water properties), and x-y-directions for C-S-H structure relaxation. The entire NPT run is carried out as a series of shorter runs, since due to the shrinkage of the simulation box the kspace mesh needs to be recalculated between the runs. Next (8-10) is the heating cycle which is necessary to overcome possible energetical barriers and reach a lower energy minimum. The timestep was lowered, since at the default 0.00028 ps the Oh-H bonds of surface silanol groups were broken. The last (12) run is the production run in which the density number of atomic species in z-direction are recorded, as well as trajectories and the radial distribution function. For more information we refer the reader to the LAMMPS input file (in.ZigaFF2)

4 Surface charge density

The surface charge density of the proposed model, shown in figure 5 of the main text, is calculated as follows. From table S11 the ionization fraction (α) at a given pH and surface silanol density (SSD) is chosen. From this the number of deprotonated silanol groups per surface area (DSG) is calculated as:

$$DSG = SSD \cdot \alpha \ [O^{-}/nm^{2}]$$

The assumption follows the adsorption of $n \cdot Ca^{2+}$ and $2(n-2) \cdot OH^{-}$ per deprotonated silanol group. Therefore, the surface charge density, σ, equals to:

$$\sigma = DSG \ [e/nm^{2}],$$

since the surface charge density without adsorbed calcium at the deprotonated silanols equals $-DSG$ [e/nm^2], and after adsorption to $+DSG$ [e/nm^2].

Table S11. Ionization fraction (α) for different surface silanol densities (4.8, 2.8 and 0.8 OH/nm^2) and pHs as predicted by Grand Canonical Monte Carlo simulations for surfaces in contact with 2 mM CaX_2 solutions[9]. X stands for any monovalent ion.

pH	α at 4.8 OH/nm^2	α at 2.8 OH/nm^2	α at 0.8 OH/nm^2
8	0	0	0
9	0.2	0.131	0.07
10	0.82	0.55	0.25
11	2.23	1.64	0.61
12	3.64	2.48	0.77
13	4.5	2.76	0.8
14	4.74	2.78	0.8

5 Calcium adsorption: Si-Surface 2

To examine Ca^{2+} adsorption at the Si-Surface 2, the tobermorite 14 Å structure with the (001) surface was investigated. We remind the reader, the basal (001) surface of tobermorite (cut through the interlayer) is the Si-Surface 2 in the present paper. The tobermorite structure was 3 layers, 2 interlayers thick, and placed in contact with a calcium-chloride solution (figure S4). The (001) surface was modeled with 90% deprotonation of silanol groups. The negative surface charge was compensated with Ca^{2+} ions (45 ions). Additional Ca^{2+} ions (8 in total) and Cl^- ions (16 ions) were placed between the surfaces. The surfaces were separated by approximately 80Å. All ions and water molecules were randomly placed between the surfaces. [25]

The inspection of the system reveals that approximately 14% of Ca^{2+} were inner sphere adsorbed. Inner sphere adsorption here is defined as silicate oxygen (protonated or deprotonated) entering the first coordination shell of Ca^{2+}, which happens when the distance between the calcium and oxygen is less than 3.15 Å.[32] The preferential outer sphere adsorption is further confirmed with the radial distribution function for calcium-oxygen (figure S5), where the first peak at approximately 2.4 Å points to inner sphere adsorption, while the intensity of the second peak at 4.7 Å points to preferred outer sphere adsorption (62% of Ca^{2+}). This can also be seen from the number density distribution along the z-axis (figure S5). 24% of Ca^{2+} remain in solution.

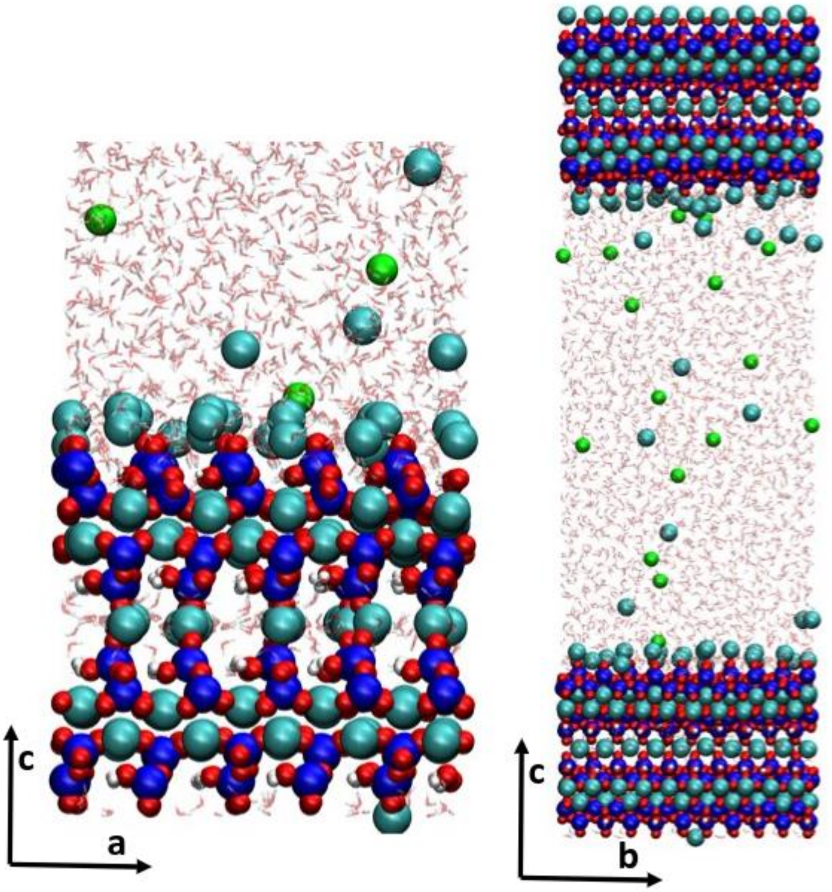

Figure S4. Final snapshot of the simulated system. Color code: Ca-cyan, Si-blue, O-red, H-white, Cl-green.

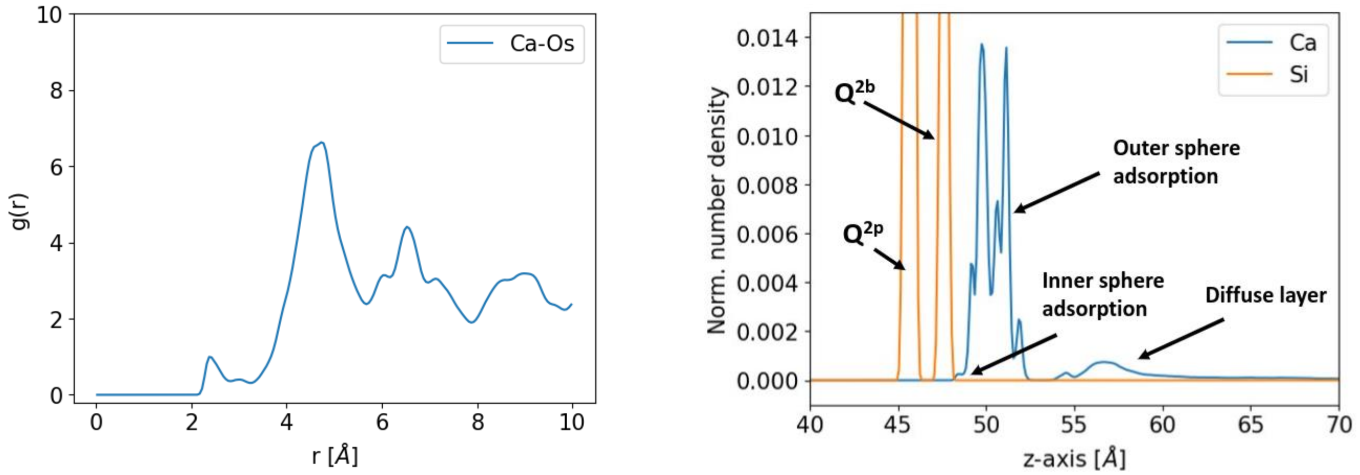

Figure S5. Left: radial distribution function for calcium-silicate oxygen. Right: Number density in z-axis for calcium and silicates. The first silicate peak belongs to Q^{2p} while the second belongs to Q^{2b} silicates.

Closer inspection of the adsorption sites reveals two mechanisms for inner sphere adsorption (figure S6). First is the adsorption on top of the Q^{2p}-Q^{2p} dimer, where Ca^{2+} inner sphere adsorbs to the sharing oxygens of the silicates and an additional deprotonated silanol group. The deprotonated silanol group can be the upper non-sharing oxygen of the next in-chain Q^{2b} (shown in figure S6b), or the lower non-sharing oxygen of the neighboring silicate chain Q^{2b}. This adsorption site was first reported by Kalinichev et al.[12], where they studied calcium adsorption on the tobermorite 9 Å (001) surface. A similar site was also observed by Kunhi Mohamed[33] in the C-S-H interlayer, who carried out a density functional theory study of the favorable calcium sites and reported this site as the energetically most favorable. The second inner sphere adsorption site (figure S6c) is due to only one silicate oxygen entering the first coordination shell of Ca^{2+}. The upper silanol group was identified as highly acidic (pKa = 6.15),[34] where the solvent contribution for deprotonation is high. Further, Androniuk and Kalinichev carried out a metadynamics adsorption study,[35] where they identified this second site as energetically preferential for Ca^{2+} adsorption.

The typical environment for outer sphere adsorption is shown in figure S6 with a triangle. Due to the structural characteristics of the tobermorite structure each next silicate chain is shifted in the b-axis direction by one silicate. Therefore, looking in the a-axis direction a Q^{2p}-Q^{2p} dimer is neighboring a Q^{2b} silicate. The upper deprotonated silanol groups of the Q^{2b} silicates (corners of the triangle in figure S6) seem to be equally attractive to the calcium ion, positioning it approximately in the geometrical center of them. There are two distinguished heights of adsorption, as seen in figure S7a and the number density along z-axis direction in figure S5. The lower height is approximately at the same height as the upper deprotonated silanol group.

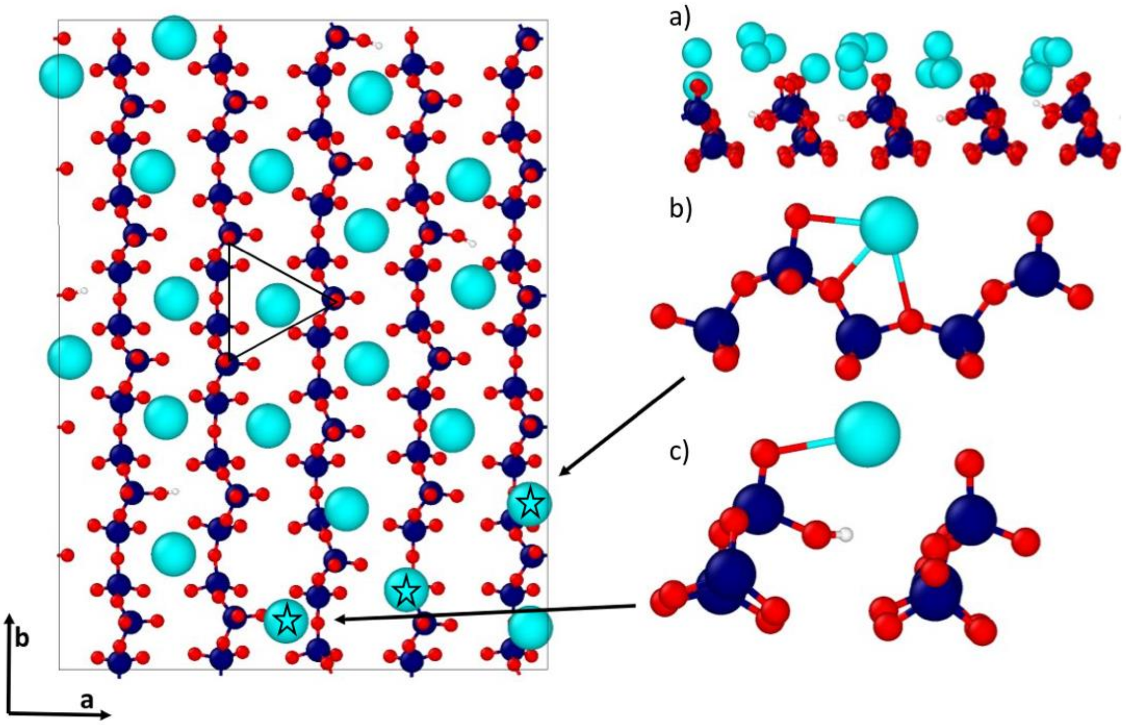

Figure S6. Location of inner (star) and outer (triangle) sphere adsorbed Ca^{2+}. Everything beside the silicates and surface calcium is hidden for clarity. First shell coordination is shown with a bond. Color code: Ca-cyan, Si-blue, O-red, H-white.

Labbez et al.[9] predicted a surface charge of 4.5 e/nm^2, for this type of surface at 90% deprotonation (pH 13) in contact with 2mM CaX_2 solution (X being any monovalent anion). Accounting for all inner and outer sphere adsorbed Ca^{2+} (below 53 Å in figure S5) the resulting surface charge of the surface equals –1.2 e/nm^2.

6 Calcium-Hydroxide Surface Network

The calcium-hydroxide network of the C-S-H (001) surface was investigated for the Ca/Si = 1.7 system, as described in the main text. To exclude possible effects of the initial configuration, two separate simulations with different initial configurations were carried out. The difference was in the initial placement of Ca^{2+} and OH^- next to the surface (see figure S7). The first (1) initial configuration had the calcium placed according to the adsorption sites in the literature.[12,33,35] As explained in the previous section, those sites are Ca^{2+} on top of the Q^{2p}-Q^{2p} (Q^1-Q^1) dimer and on top of the Q^{2b} silicate. The OH^- were placed in the proximity of the Individual calcium ions. The second (2) configuration had the same Mixed-Surface with Ca_B and Q^{2b} termination, although the required Ca^{2+} and OH^- were randomly placed in the 30 Å slab next to the surface. The ions, as well as the water molecules were inserted using Packmol.[25]

In both cases a similar calcium-hydroxide networks formed on the surface (figure S9). Both networks started to form in a similar manner, therefore only the first (1) initial configuration will be examined in the following discussion. The final configuration data files for both simulations are provided as part of the SI.

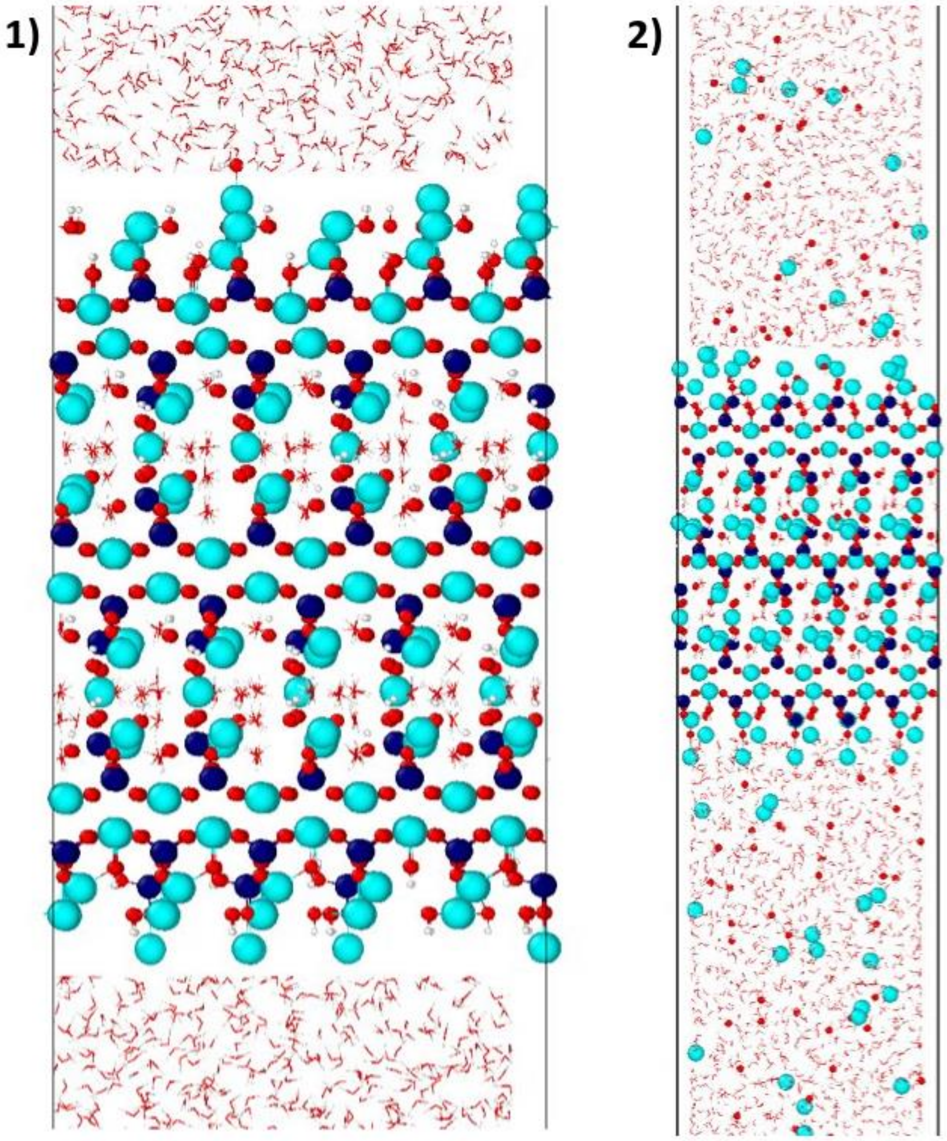

Figure S7. Initial configuration of the C-S-H nanofoil: 1) Ca^{2+} located at the dimer and Q^{2b} adsorption sites and 2) Ca^{2+} located on top of the deprotonated silanol groups, the remaining Ca^{2+} and OH^{-} are randomly inserted next to the surface. Color code: Ca-cyan, Si-blue, O-red, H-white.

As seen in figure S8, The Ca-O radial distribution function (rdf) shows the affinity of Ca^{2+} for inner sphere adsorption to the deprotonated silanol groups (O). The high intensity peak for the Mixed-Surface with Ca^{2+} and OH^{-} adsorption, at approximately 2.3 Å, clearly points to a preferred inner sphere adsorption in comparison to the Si-Surface 2 with Ca^{2+} adsorption. The higher packing density of Ca^{2+} next to the surface is further confirmed with the Ca-Ca rdf. The Ca-OH network of the Mixed-Surface results in Ca-Ca distance of around 4 Å, while the pure Ca^{2+} adsorption on the Si-Surface results in Ca-Ca distances of roughly 7 Å.

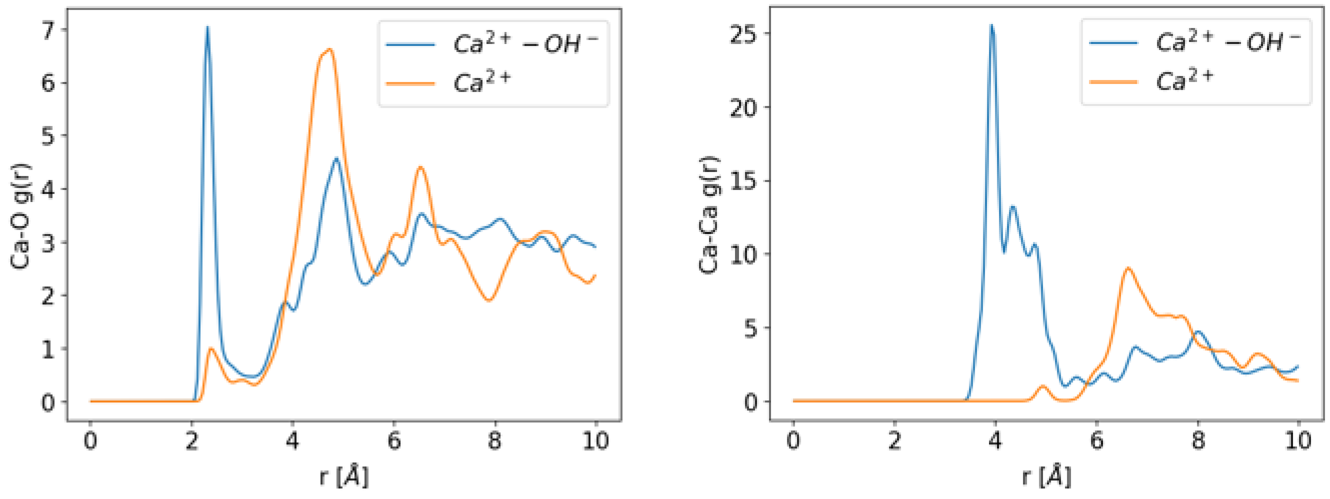

Figure S8. Comparison of Ca-O and Ca-Ca radial distribution functions for the Si-Surface2 with Ca^{2+} adsorption (orange) and the Mixed-Surface with Ca^{2+} and OH^- adsorption (blue).

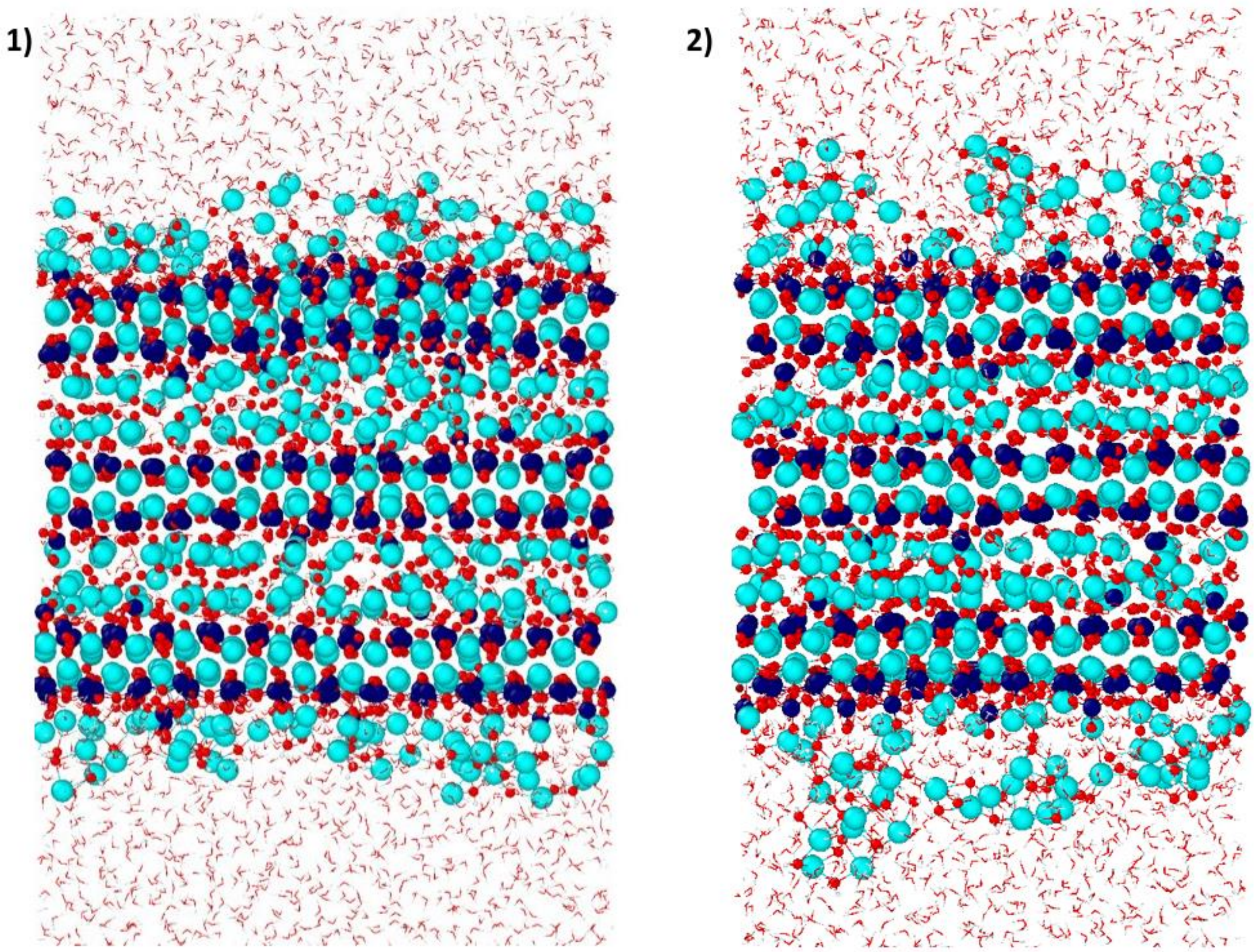

Figure S9. Final configuration of the C-S-H nanofoil shown in figure S6. Color code: Ca-cyan, Si-blue, O-red, H-white.

Excluding Ca_B (calcium in the bridging site) 30% of the Ca^{2+} are inner sphere adsorbed. Again, here we refer to inner sphere adsorption if a Ca^{2+} is less than 3.15 Å away from a silicate oxygen (first shell coordination). Including Ca_B

roughly 60 % of calcium at the surface is inner sphere adsorbed. As can be seen in figures S10 and S11, the inner sphere adsorption is exclusively associated with the adsorption on top of the Q^1-Q^1 (Q^{2p}-Q^{2p}) dimer, which was previously observed by Kalinichev et al.[12] The adsorption at the dimer site is further favored when the neighboring silicate chain carries the Q^{2b} species. However, in 50 % of the inner sphere adsorptions (Ca_B excluded) a hydroxide (OH^-) is acting additionally as a bridge between the adsorbed Ca^{2+} and the main layer calcium (figure S11, black arrow).

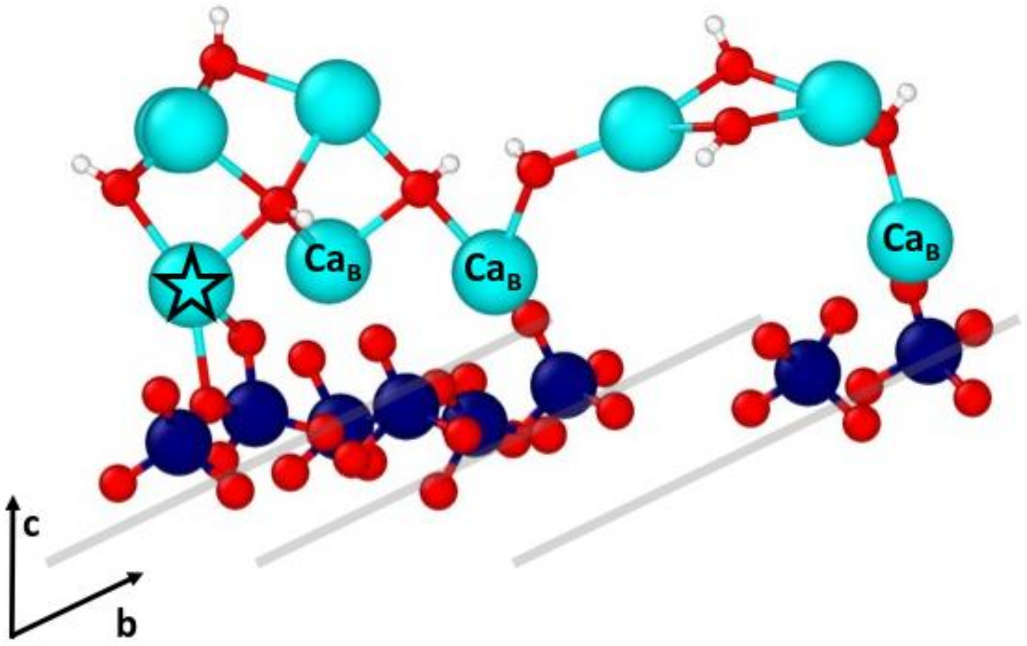

Figure S10. Formation of the calcium-hydroxide network. Ca_B corresponds to the calcium in the bridging site. The star marks the inner sphere adsorbed calcium. Gray lines show the direction of the silicate chains (b-axis). Color code: Ca-cyan, Si-blue, O-red, H-white.

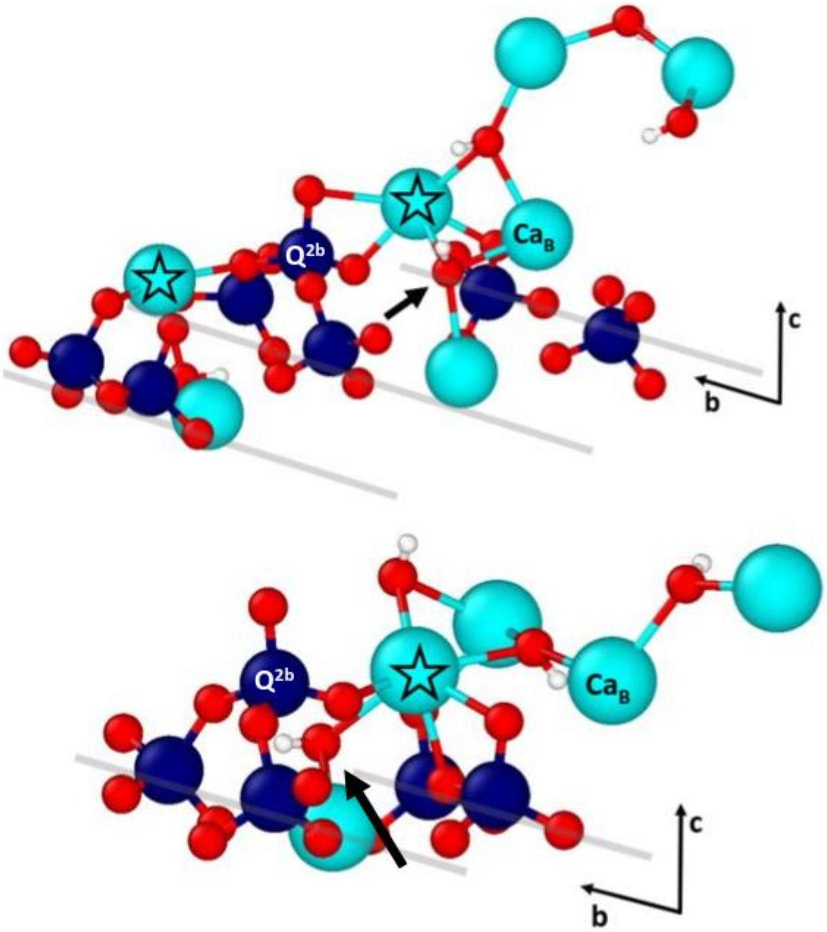

Figure S11. Typical inner sphere adsorption of calcium ions and the start of the calcium-hydroxide network. The star marks the inner sphere adsorbed calcium, Ca_B is calcium in the bridging site (linking dimers), and the arrow points to the hydroxide on the main layer calcium chains, which acts as a bridge for calcium adsorption. Gray lines show the direction of the silicate chains (b-axis). Color code: Ca-cyan, Si-blue, O-red, H-white.

References

(1) Kunhi Mohamed, A.; Parker, S. C.; Bowen, P.; Galmarini, S. An Atomistic Building Block Description of C-S-H - Towards a Realistic C-S-H Model. *Cem Concr Res* **2018**, *107* (March), 221–235. https://doi.org/10.1016/j.cemconres.2018.01.007.

(2) Předota, M.; Machesky, M. L.; Wesolowski, D. J. Molecular Origins of the Zeta Potential. *Langmuir* **2016**, *32* (40), 10189–10198. https://doi.org/10.1021/acs.langmuir.6b02493.

(3) Masoumi, S.; Zare, S.; Valipour, H.; Abdolhosseini Qomi, M. J. Effective Interactions between Calcium-Silicate-Hydrate Nanolayers. *Journal of Physical Chemistry C* **2019**, *123* (8), 4755–4766. https://doi.org/10.1021/acs.jpcc.8b08146.

(4) Heinz, O.; Heinz, H. Cement Interfaces: Current Understanding, Challenges, and Opportunities. *Langmuir* **2021**, *37* (21), 6347–6356. https://doi.org/10.1021/acs.langmuir.1c00617.

(5) Shishehbor, M.; Sakaniwa, D.; Stefaniuk, D.; Krakowiak, K. J.; Abdolhosseini Qomi, M. J. On the Significance of Interfacial Chemistry on the Strength of Fly Ash-Cement Composites. *Cem Concr Res* **2022**, *151* (October 2021), 106619. https://doi.org/10.1016/j.cemconres.2021.106619.

(6) Deng, H.; He, Z. Interactions of Sodium Chloride Solution and Calcium Silicate Hydrate with Different Calcium to Silicon Ratios: A Molecular Dynamics Study. *Constr Build Mater* **2021**, *268*, 121067. https://doi.org/10.1016/j.conbuildmat.2020.121067.

(7) Hong, S. Y.; Glasser, F. P. Alkali Binding in Cement Pastes : Part I. The C-S-H Phase. *Cem Concr Res* **1999**, *29* (12), 1893–1903. https://doi.org/10.1016/S0008-8846(99)00187-8.

(8) Glasser, F. P. Characterisation of the Barrier Performance of Cements. In *Materials Research Society Symposium - Proceedings*; Materials Research Society, 2002; Vol. 713, pp 721–732. https://doi.org/10.1557/proc-713-jj9.1.

(9) Labbez, C.; Jönsson, B.; Pochard, I.; Nonat, A.; Cabane, B. Surface Charge Density and Electrokinetic Potential of Highly Charged Minerals: Experiments and Monte Carlo Simulations on Calcium Silicate Hydrate. *Journal of Physical Chemistry B* **2006**, *110* (18), 9219–9230. https://doi.org/10.1021/jp057096+.

(10) Yoshida, S.; Elakneswaren, Y.; Nawa, T. Electrostatic Properties of C-S-H and C-A-S-H for Predicting Calcium and Chloride Adsorption. *Cem Concr Compos* **2021**, *121* (104109).

(11) Marry, V.; Rotenberg, B.; Turq, P. Structure and Dynamics of Water at a Clay Surface from Molecular Dynamics Simulation. *Physical Chemistry Chemical Physics* **2008**, *10* (32), 4676–4677. https://doi.org/10.1039/b812223g.

(12) Kalinichev, A. G.; Wang, J.; Kirkpatrick, R. J. Molecular Dynamics Modeling of the Structure, Dynamics and Energetics of Mineral–Water Interfaces: Application to Cement Materials. **2007**, *37*, 337–347. https://doi.org/10.1016/j.cemconres.2006.07.004.

(13) Duque-Redondo, E.; Bonnaud, P. A.; Manzano, H. A Comprehensive Review of C-S-H Empirical and Computational Models, Their Applications, and Practical Aspects. *Cem Concr Res* **2022**, *156*, 106784. https://doi.org/10.1016/j.cemconres.2022.106784.

(14) Kumar, A.; Walder, B. J.; Kunhi Mohamed, A.; Hofstetter, A.; Srinivasan, B.; Rossini, A. J.; Scrivener, K.; Emsley, L.; Bowen, P. The Atomic-Level Structure of Cementitious Calcium Silicate Hydrate. *The Journal of Physical Chemistry C* **2017**, *121* (32), 17188–17196. https://doi.org/10.1021/acs.jpcc.7b02439.

(15) Chen, J. J.; Thomas, J. J.; Taylor, H. F. W.; Jennings, H. M. Solubility and Structure of Calcium Silicate Hydrate. *Cem Concr Res* **2004**, *34* (9), 1499–1519. https://doi.org/10.1016/j.cemconres.2004.04.034.

(16) Abdolhosseini Qomi, M. J.; Krakowiak, K. J.; Bauchy, M.; Stewart, K. L.; Shahsavari, R.; Jagannathan, D.; Brommer, D. B.; Baronnet, A.; Buehler, M. J.; Yip, S.; Ulm, F. J.; Van Vliet, K. J.; Pellenq, R. J. M. Combinatorial Molecular Optimization of Cement Hydrates. *Nat Commun* **2014**, *5*, 1–10. https://doi.org/10.1038/ncomms5960.

(17) Maddalena, R.; Li, K.; Chater, P. A.; Michalik, S.; Hamilton, A. Direct Synthesis of a Solid Calcium-Silicate-Hydrate (C-S-H). *Constr Build Mater* **2019**, *223*, 554–565. https://doi.org/10.1016/j.conbuildmat.2019.06.024.

(18) Yan, Y.; Yang, S.-Y.; Miron, G. D.; Collings, I. E.; L'Hôpital, E.; Skibsted, J.; Winnefeld, F.; Scrivener, K.; Lothenbach, B. Effect of Alkali Hydroxide on Calcium Silicate Hydrate (C-S-H). *Cem Concr Res* **2022**, *151*, 106636. https://doi.org/10.1016/j.cemconres.2021.106636.

(19) Cong, X.; Kirkpatrick, R. J. 17O MAS NMR Investigation of the Structure of Calcium Silicate Hydrate Gel. *Journal of the American Ceramic Society* **1996**, *79* (6), 1585–1592. https://doi.org/10.1111/j.1151-2916.1996.tb08768.x.

(20) Yu, P.; Kirkpatrick, R. J.; Poe, B.; McMillan, P. F.; Cong, X. Structure of Calcium Silicate Hydrate (C-S-H): Near-, Mid-, and Far-Infrared Spectroscopy. *Journal of the American Ceramic Society* **2004**, *82* (3), 742–748. https://doi.org/10.1111/j.1151-2916.1999.tb01826.x.

(21) Cong, X.; James Kirkpatrick, R. 29Si MAS NMR Study of the Structure of Calcium Silicate Hydrate. *Advanced Cement Based Materials* **1996**, *3* (3–4), 144–156. https://doi.org/10.1016/s1065-7355(96)90046-2.

(22) Thomas, J. J.; Chen, J. J.; Jennings, H. M.; Neumann, D. A. Ca-OH Bonding in the C-S-H Gel Phase of Tricalcium Silicate and White Portland Cement Pastes Measured by Inelastic Neutron Scattering. *Chemistry of Materials* **2003**, *15* (20), 3813–3817. https://doi.org/10.1021/cm034227f.

(23) Morales-Melgares, A.; Casar, Z.; Moutzouri, P.; Venkatesh, A.; Cordova, M.; Kunhi Mohamed, A.; L. Scrivener, K.; Bowen, P.; Emsley, L. Atomic-Level Structure of Zinc-Modified Cementitious Calcium Silicate Hydrate. *J Am Chem Soc* **2022**, *144* (50), 22915–22924. https://doi.org/10.1021/jacs.2c06749.

(24) Momma, K.; Izumi, F. VESTA: A Three-Dimensional Visualization System for Electronic and Structural Analysis. *J Appl Crystallogr* **2008**, *41* (3), 653–658. https://doi.org/10.1107/S0021889808012016.

(25) Martinez, L.; Andrade, R.; Birgin, E. G.; Martínez, J. M. PACKMOL: A Package for Building Initial Configurations for Molecular Dynamics Simulations. *J Comput Chem* **2009**, *30* (13), 2157–2164. https://doi.org/10.1002/jcc.21224.

(26) Valavi, M.; Casar, Z.; Kunhi, A.; Bowen, P.; Galmarini, S. Molecular Dynamic Simulations of Cementitious Systems Using a Newly Developed Force Field Suite ERICA FF. *Cem Concr Res* **2022**, *154*, 106712. https://doi.org/10.1016/j.cemconres.2022.106712.

(27) Kunhi Mohamed, A.; Moutzouri, P.; Berruyer, P.; Walder, B. J.; Siramanont, J.; Harris, M.; Negroni, M.; Galmarini, S. C.; Parker, S. C.; Scrivener, K. L.; Emsley, L.; Bowen, P. The Atomic-Level Structure of Cementitious Calcium Aluminate Silicate Hydrate. *J Am Chem Soc* **2020**, *142* (25), 11060–11071. https://doi.org/10.1021/jacs.0c02988.

(28) Mishra, R. K.; Mohamed, A. K.; Geissbühler, D.; Manzano, H.; Jamil, T.; Shahsavari, R.; Kalinichev, A. G.; Galmarini, S.; Tao, L.; Heinz, H.; Pellenq, R.; van Duin, A. C. T.; Parker, S. C.; Flatt, R. J.; Bowen, P. Cemff: A Force Field Database for Cementitious Materials Including Validations, Applications and Opportunities. *Cem Concr Res* **2017**, *102*, 68–89. https://doi.org/10.1016/j.cemconres.2017.09.003.

(29) Abascal, J. L. F.; Vega, C. A General Purpose Model for the Condensed Phases of Water: TIP4P/2005. *Journal of Chemical Physics* **2005**, *123* (23). https://doi.org/10.1063/1.2121687.

(30) Thompson, A. P.; Aktulga, H. M.; Berger, R.; Bolintineanu, D. S.; Brown, W. M.; Crozier, P. S.; in 't Veld, P. J.; Kohlmeyer, A.; Moore, S. G.; Nguyen, T. D.; Shan, R.; Stevens, M. J.; Tranchida, J.; Trott, C.; Plimpton, S. J. LAMMPS - a Flexible Simulation Tool for Particle-Based Materials Modeling at the Atomic, Meso, and Continuum Scales. *Comput Phys Commun* **2022**, *271*, 108171. https://doi.org/10.1016/j.cpc.2021.108171.

(31) Mitchell, P. J.; Fincham, D. Shell Model Simulations by Adiabatic Dynamics. *Journal of Physics: Condensed Matter* **1993**, *5*, 1031–1038. https://doi.org/10.1088/0953-8984/5/8/006.

(32) Bischoff, M.; Biriukov, D.; Předota, M.; Marchioro, A. Second Harmonic Scattering Reveals Ion-Specific Effects at the SiO2 and TiO2 Nanoparticle/Aqueous Interface. *Journal of Physical Chemistry C* **2021**, *125* (45), 25261–25274. https://doi.org/10.1021/acs.jpcc.1c07191.

(33) Kunhi Mohamed, A. Atomistic Simulations of The Structure of Calcium Silicate Hydrates: Interlayer Positions, Water Content and a General Structural Brick Model, EPFL, 2018, Vol. TH 8840. https://doi.org/10.5075/EPFL-THESIS-8840.

(34) Churakov, S. v.; Labbez, C.; Pegado, L.; Sulpizi, M. Intrinsic Acidity of Surface Sites in Calcium Silicate Hydrates and Its Implication to Their Electrokinetic Properties. *Journal of Physical Chemistry C* **2014**, *118* (22), 11752–11762. https://doi.org/10.1021/jp502514a.

(35) Androniuk, I.; Kalinichev, A. G. Molecular Dynamics Simulation of the Interaction of Uranium (VI) with the C–S–H Phase of Cement in the Presence of Gluconate. *Applied Geochemistry* **2020**, *113* (July 2019), 104496. https://doi.org/10.1016/j.apgeochem.2019.104496.
